# Patient characteristics and clinical determinants of outpatient red cell transfusions

**DOI:** 10.1111/trf.18428

**Published:** 2025-10-15

**Authors:** Nora Hemesath, Daniel Fürst, Marianne Holl, Bianca Ries, Astrid Marx‐Hofmann, Christine Kroll, Britta Höchsmann, Bernd Jahrsdörfer, Christof Weinstock, Hubert Schrezenmeier, Sixten Körper

**Affiliations:** ^1^ Institute for Clinical Transfusion Medicine and Immunogenetics Ulm, German Red Cross Blood Transfusion Service Baden‐Württemberg‐Hessen and University Hospital Ulm and Institute of Transfusion Medicine University of Ulm Ulm Germany

**Keywords:** chronic anemia, outpatients, red cell transfusion

## Abstract

**Background:**

There is a knowledge gap in the outpatient transfusion practice of red blood cells (RBC) according to optimal trigger, dose, and interval as well as which patients are in need of outpatient transfusion.

**Study Design and Methods:**

This single‐center retrospective analysis investigates RBC transfusion practices in 804 patients with 5591 visits.

**Results:**

Blood disorders and solid tumors were the primary indications for transfusion, accounting for 82% of RBC use. Median (interquartile range) pretransfusion hemoglobin (Hb) level was 7.5 (6.9;8.1)g/dL. Linear regression models revealed that Hb increased by 1.04 g/dL per unit transfused and identified the concomitant transfusion of platelets, male sex, higher height, weight, and lower pre‐transfusion Hb level as negative factors for the increase in Hb. Factors that correlate with Hb decline after transfusion include lower reticulocytes, male sex, height, and concomitant platelet transfusion. A longer interval to next transfusion was associated with higher reticulocyte counts, higher pre‐transfusion Hb levels and number of units transfused. According to our models, the potential for reducing the use of RBC through single‐unit transfusions decreases with increasing height, concomitant platelet transfusions, and lower pretransfusion Hb levels and is going along with a shorter interval to next transfusion. Nonsevere adverse reactions occurred in ≤0.24% of transfusions.

**Conclusion:**

These results highlighted the effect of patient age, sex, Hb, height, and weight on outpatient RBC transfusion. These variables have to be considered in clinical practice and trials evaluating patient outcomes.

**Abbreviations**
AA/PNH: aplastic anemia/paroxysmal nocturnal hemoglobinuria
AML: acute myeloid leukemia
A‐NOS: anemia not otherwise specified
AST: antibody screening test
BD: blood disorders
FFP: fresh frozen plasma
GFR: glomerular filtration rate
Hb: hemoglobin
IQR: interquartile range
MDS: myelodysplastic syndromes
ml: milliliters
MPN: myeloproliferative neoplasms
OR: odds ratio
PLT: platelet
RBC: red blood cell
ROC: receiver‐operating‐characteristics
SD: standard deviation
ST: solid tumor

## 1 INTRODUCTION

Red cell transfusion in the outpatient setting is performed to maintain organ function and quality of life.^1^, ^2^, ^3^ Most of the literature on red blood cell (RBC) transfusions focuses on inpatients with acute anemia, addressing transfusion triggers, safety, and survival.^4^, ^5^, ^6^, ^7^, ^8^, ^9^, ^10^ Current German cross‐sectional guidelines, as well as other international guidelines, recommend a restrictive approach to blood transfusion at hemoglobin levels of 7–7.5 g/dL for inpatients.^6^, ^11^ In line with the restrictive management of blood products, the administration of a single RBC unit is generally preferred^12^, ^13^ and investigated in some trials with hematological patients.^14^, ^15^

Data on RBC transfusions in the outpatient setting are available for patients with kidney disease and myelodysplastic syndromes.^1^, ^16^, ^17^ However, data on the range of diseases leading to transfusion in the outpatient context remain scarce. Currently, there is limited information on patients with bone marrow failure syndromes (e.g., aplastic anemia), chronic hematological disorders (e.g., myelodysplastic syndromes, osteomyelofibrosis), and solid tumors (ST), who often receive chronic transfusions in the outpatient setting.^2^

This retrospective analysis aims to provide a comprehensive overview of current practices in outpatient red blood cell (RBC) transfusions, focusing on the patient population, diagnostic spectrum, age and sex distribution, as well as the number of RBC transfusions and associated complications during the analyzed period. The study investigated the hypothesis that underlying disorders, comorbidities, cardiac function (measured by pro‐NT‐BNP), renal function, age, and distance from the transfusion center have an influence on transfusion triggers and the number of RBC units transfused. Furthermore, we hypothesized that the increase in hemoglobin levels is determined by the underlying disease causing anemia, concomitant thrombocytopenia, sex, and age.

Thus our results provide a basis for the design of further prospective clinical trials in the context of outpatient transfusion.

## 2 STUDY DESIGN AND METHODS

### 2.1 Design and patients

This is a single‐center retrospective analysis from a blood bank with a large outpatient transfusion center. Inclusion criteria required one visit at our outpatient clinic with a blood cell count in the year 2021 or 2022. These broad inclusion criteria were chosen to provide a comprehensive description of the patients with the need for an outpatient transfusion. Any patient with visits in 2021 or 2022 was included in the analysis (*n* = 804); from these patients, visits in our institution were included back to the year 2018. Data were retrieved from patient records for each visit, regardless of transfusion status. Patients may have received transfusions at other institutions during the study period that are not included in this analysis.

### 2.2 Transfusion

From August 2018 on, red cell concentrates were transfused with the following specifications: volume: 225–385 mL, hematocrit: 0.5–0.7 L/L, total hemoglobin ≥40 g/unit, leukocytes <1 × 10^6^/unit, additive solution: 0.29–0.4 mL/mL PAGGS‐M (phosphate‐adenine‐glucose‐guanosine‐saline mannitol).^18^ Before August 2018, RBC products with the additive solution SAG‐M were used.

RBC transfusions followed German cross‐sectional guidelines,^19^ with a transfusion trigger of <8 g/dL based on physician discretion, taking symptoms into account. Some patients received follow‐up appointments at the transfusion clinic, while others were referred by their attending physician as needed. Crossmatching was performed the same day or up to 3 days before transfusion.

### 2.3 Statistical analysis

Data were transferred from paper records into Microsoft Excel for basic analysis (e.g., filtering, calculations of Hb differences, body surface area, logical functions). Further statistical analysis was conducted in GraphPad Prism (version 9; GraphPad Software, LLC, Boston, USA). Data are presented as medians with interquartile ranges unless otherwise specified. The Mann–Whitney test was used for unpaired comparisons. The Kruskal‐Wallis test with Dunn's test for correction of multiple comparisons was used for comparisons across more than two groups. Statistical significance is indicated by *p < .05; lower p‐values are denoted by **p < .01, ***p < .001, ****p < .0001.

The transfusion trigger was represented and operationalized by the calculation of an odds ratio for RBC transfusion. More details are provided in the supplement.

## 3 RESULTS

### 3.1 Outpatient population

This retrospective analysis included 804 patients with 5591 visits in the years 2021 or 2022 (Table 1). One hundred sixty‐four out of 804 patients (20.4%) had previous transfusions in our outpatient clinic. For these patients, an additional 2233 visits were documented back to 2018 (Table S2).

**TABLE 1:** Patients and visits.

	Total *N* = 804 (100%)	BD *N* = 365 (46%)	Solid tumor *N* = 228 (28%)	A‐NOS *N* = 137 (17%)	Other *N* = 74 (9%)	BD vs. ST	A‐NOS vs. BD	A‐NOS vs. ST
Demographic and clinical characteristics								
Gender, no (%)								
Female	350 (44)	143 (39)	93 (41)	81 (60)	33 (44)	–	–	–
Male	454 (56)	222 (61)	135 (59)	55 (40)	42 (56)			
Median age, years (IQR)	72.6 (61.6–81.7)	69.6 (58.5–78.9)	69.9 (61.5–78.5)	82.1 (73.8–7.8)	81.5 (70.6–88.1)	ns	****	****
Heart disease, no (%)	248 (30.8)	87 (23.8)	53 (23.2)	70 (51.5)	38 (50.7)	–	–	–
RBC transfusion only, no (%)	560 (70)	171 (47)	192 (84)	129 (94)	68 (92)	–	–	–
PLT transfusion only, no (%)	50 (6)	42 (12)	5 (2)	1 (0.7)	2 (3)	–	–	
Patients with PLT and RBC transfusion, no (%)	180 (22)	142 (39)	29 (13)	7 (5)	2 (3)	–	–	–
FFP, no	2	2	0	0	0	–	–	–
No transfusion, no	14	10	2	0	2	–	–	–
Traveldistance, km (IQR)	17.0 (10.0–32)	18.0 (10.0–45.0)	18.0 (10.3–32.8)	14.0 (8.0–21.0)	18.0 (14.0–24.0)	ns	**	*
Iron chelator, no (%)	29 (3.6)	28 (7.6)	0 (0)	1 (0.7)	0 (0)	–	–	–
Patients before 2021, no	164	98	28	22	16	–	–	–
Visits 2021/2022	5591 (100%)	4285 (76%)	653 (12%)	433 (8%)	220 (4%)	BD vs. ST	A‐NOS vs. BD	A‐NOS vs. ST
Gender, no (%)								
Female	2117 (38)	1500 (35)	299 (46)	222 (51)	96 (44)			
Male	3474 (62)	2785 (65)	354 (54)	211 (49)	124 (56)	–	–	–
Median age, years (IQR)	71.4 (62.7–79.6)	70.4 (61.1–78.8)	67.3 (58.8–74.6)	81.8 (72.6–88.4)	84.1 (73.3–91.6)	***	****	****
Heart disease, no (%)	1625 (29.1)	1080 (25.2)	162 (24.8)	258 (59.6)	125 (56.8)	–	–	–
Symptoms no (%)								
Dyspnea	1049 (18.8)	593 (13.8)	198 (30.3)	189 (43.6)	69 (31.4)	–	–	–
Fatigue	2223 (39.8)	1410 (32.9)	400 (61.3)	284 (65.6)	129 (58.6)	–	–	–
Visits with RBC Transfusion, no	3277	2216	519	355	187			
Visits with 1 RBC transfusion	879	732	56	47	44			
Visits with 2 RBC transfusion	2397	1484	463	307	143			
Visits with 3 RBC transfusion	1	0	0	1	0	–	–	–
RBC transfusion, units	5676	3700	982	664	330	****	****	ns
Plt transfusion, units	2424	2286	104	30	4	ns	ns	ns
Neutrophiles, 10^3^/μL (IQR)	2.7 (0.9–5.4)	2.0 (0.7–5.1)	3.9 (2.4–6.3)	3.7 (2.3–5.1)	4.1 (3.0–5.4)	****	****	ns
Hemoglobin, g/dL (IQR)	8.1 (7.3–9)	8.2 (7.4–9.1)	7.8 (7.2–8.6)	7.5 (6.6–8.3)	7.8 (7.0–8.6)	****	****	***
Reticulocytes, 10^6^/μL (IQR)	0.047 (0.017–0.086)	0.040 (0.014–0.080)	0.062 (0.033–0.095)	0.065 (0.044–0.102)	0.079 (0.057–0.101)	****	****	ns
Plt, 10^3^/μL (IQR)	34 (14–188)	23 (12–104)	153 (44–290)	217 (134–312)	169 (110–246)	****	****	****
GFR, mL/min (IQR)	65.7 (43.7–87.2)	66.6 (45.3–86.5)	76.4 (58.2–105.2)	53.8 (35.0–72.1)	37.2 (31.3–50.2)	****	****	****
Ferritin, μg/L (IQR)	1383 (438.8–3535.0)	1879 (788.4–4247.0)	797.3 (199.9–1797.0)	70.0 (15.5–1061.0)	79.4 (30.0–166.5)	****	****	****
Pro‐NT‐BNP, ng/L (IQR)	630.7 (263.7–2128.0)	630.7 (263.7–2233.0)	483.3 (221.4–1168.0)	1051.0 (386.7–2999.0)	1267.0 (742.0–3646.0)	***	****	****

*Note*: Statistical significance is indicated by *p < .05; lower *p*‐values are denoted by **p < .01, ***p < .001, ****p < .0001.

Most patients had blood disorders (BD) (46%), followed by solid tumors (ST) (28%), and anemia not otherwise specified (A‐NOS) (17%). The remaining patients, including those with gastrointestinal bleeding and renal failure, comprised 9% (Table 1). Among BD patients, the most common diagnoses were acute myeloid leukemia (AML) (25.2%), lymphoma (20.8%), myeloproliferative neoplasms (MPN) (16.9%), and myelodysplastic syndromes (MDS) (16.4%). The highest numbers of RBC units were administered to those with MDS (29.5%), AML (21.2%), MPN (16.0%), lymphoma (10.3%), and aplastic anemia or paroxysmal nocturnal hemoglobinuria (AA/PNH) (8.0%) (Figure S1). Prostate (21.3%), pulmonary (18.3%), esophageal/gastric (11.5%), and colorectal cancers (10.5%) were the most common solid tumors. Patients with BD and ST accounted for 88% of visits and received 82% of RBC units (Figure S1).

The median age was 72.6 years (range 18.6–99.3 years). Most patients were male (56%), though the A‐NOS group had a female predominance (60%).

Thirty‐one percent of patients had concomitant heart disease. Dyspnea or fatigue was reported in 367 (45.9%) and 369 (45.9%) patients, respectively. Fatigue was noted in 39.8% and dyspnea in 18.8% of visits. A positive antibody screening test (AST) was seen in 8.3% of patients and iron chelators were prescribed in a subset of 3.6% of patients who received RBC transfusions (Table 1).

### 3.2 Pre‐transfusion hemoglobin levels

**FIGURE 1:**
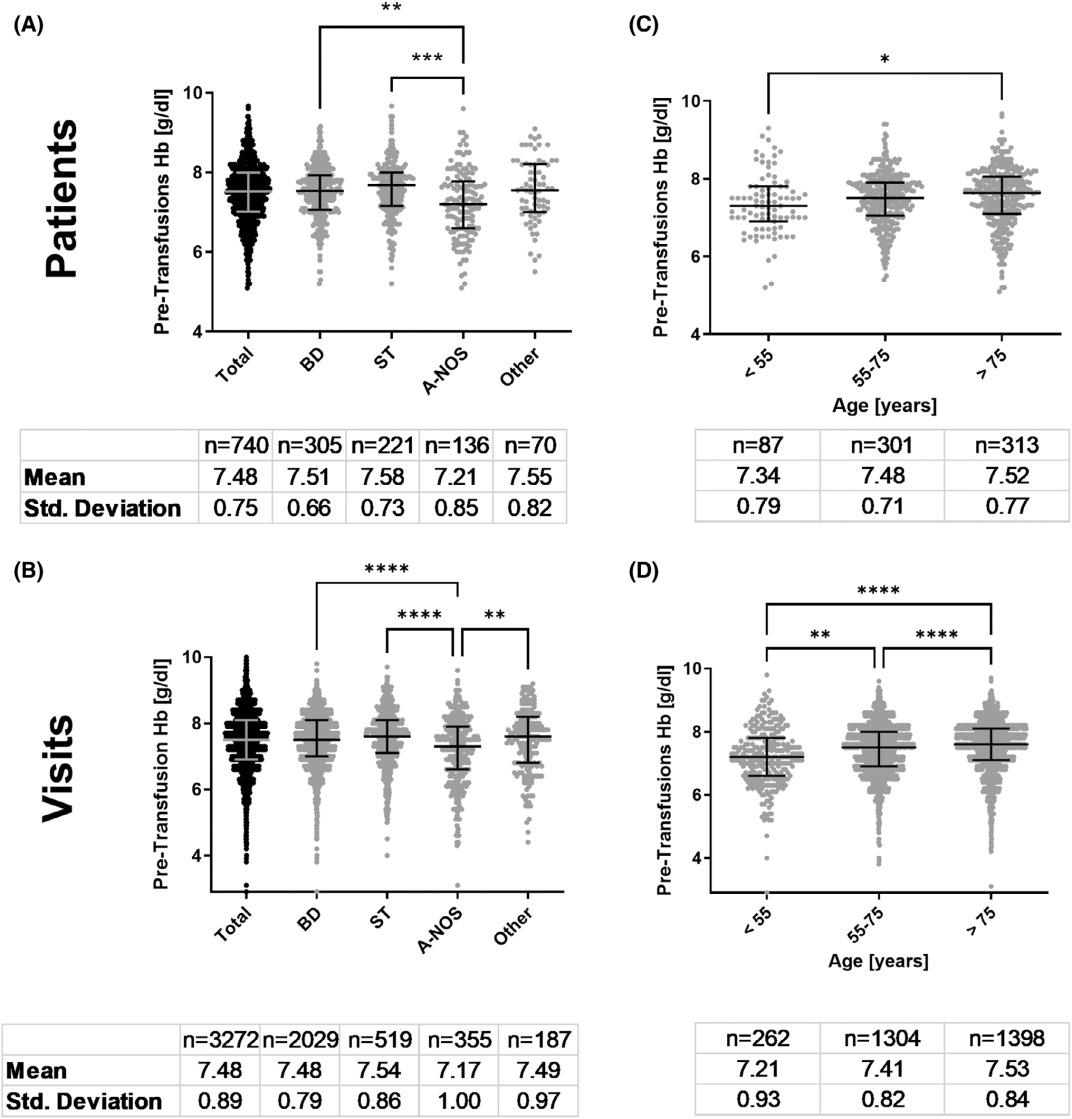
Pre‐transfusion Hb levels according to diagnosis (A,B) and age (C,D). The group of blood disorders was analyzed without patients with hemoglobinopathies. A and C show medians of the means of pre‐transfusion Hb levels of each patient. Means were calculated for each patient for all available transfusion episodes (2018–2022); in cases where only one transfusion was documented, this value was included in the analysis. B and D show the pre‐transfusion Hb levels of each transfusion visit in the years 2021–2022. Analysis was performed without patients with gastrointestinal bleeding or hemoglobinopathies. Kruskal‐Wallis test with multiple comparisons was performed for each group. Only significant tests (*p* < 0.05) were shown. The group of blood disorders was analyzed without patients with hemoglobinopathies. Statistical significance is indicated by *p < .05; lower *p*‐values are denoted by **p < .01, ***p < .001, ****p < .0001.

Figure 1 presents pre‐transfusion hemoglobin (Hb) levels across different patient groups. Panel A summarizes the mean pre‐transfusion values per patient, while panel B shows the levels per visit. The patient‐related median pre‐transfusion Hb was 7.5 g/dL, equal to the visit‐related median of 7.5 g/dL. Patients with A‐NOS had significantly lower pre‐transfusion Hb (7.2 g/dL) compared to those with BD (7.5 g/dL) or ST (7.7 g/dL).

Pre‐transfusion Hb levels by age group, ranging from 7.3 g/dL in those under 55 to 7.6 g/dL in those over 75 years (Figure 1C,D). The levels by visit showed comparable results (Figure 1D).

The differences of pre‐transfusion Hb levels between males and females were approximately 0.1 g/dL (Figure S2A–C) and were even less pronounced for the presence or absence of fatigue, dyspnea, or heart disease (Figure S2D,E).

**FIGURE 2:**
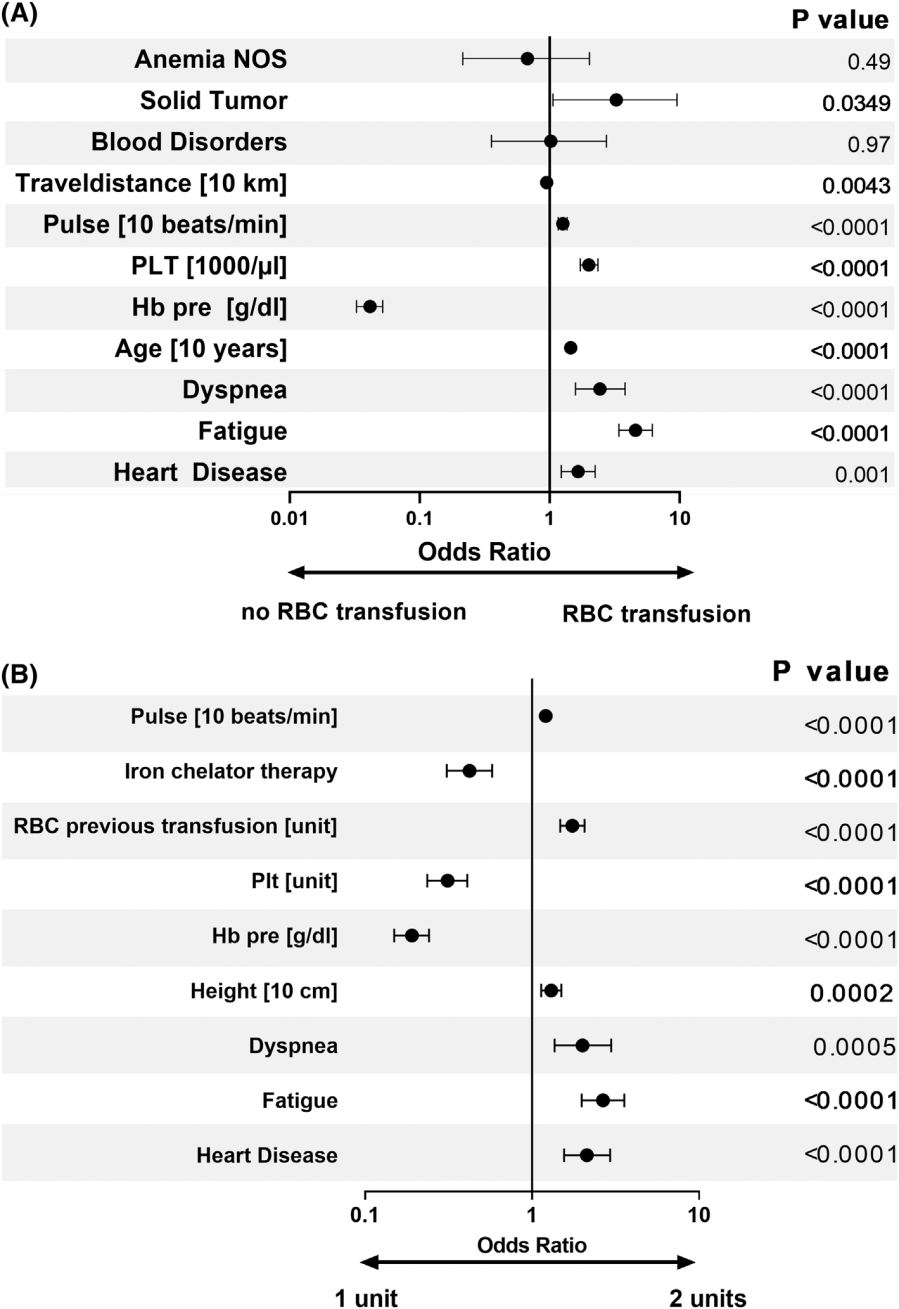
Odds Ratio for RBC transfusion (A) and one versus two RBC units (B). All visits in 2021 and 2022 were included. Patients with gastrointestinal bleeding and hemoglobinopathies were excluded. Estimates were shown with 95% confidence intervalls. (A) Odds ratio for transfusion. Visits without transfusion (e.g. blood draw for crossmatching therefore visits up to 3 days prior to transfusion were excluded from the analysis). *N* = 4306 visits were analyzed. (B) Odds ratio for two RBC units versus only one RBC unit (*N* = 1505 visits were analyzed) (see also Figure S6 for receiver‐operating‐characteristics (ROC) for RBC transfusion and one versus two RBC units).

The setting of transfusion trigger was investigated in terms of factors influencing the decision to administer a RBC transfusion; we calculated the Odds Ratios (OR) for each visit, indicating the likelihood of a transfusion (OR >1) or its avoidance (OR <1). Pre‐transfusion Hb had the strongest impact, significantly reducing the odds of transfusion (OR = 0.04 per g/dL Hb). Symptoms like fatigue (OR = 4.57), dyspnoea (OR = 2.43), and heart disease (OR = 1.65) increased the likelihood of transfusion. An increase in heart rate raised the OR by 1.26 per 10 beats/min. Travel distance to the clinic reduced the OR by 0.95 per 10 km. Age increased the OR by 1.45 per decade, and platelet count raised the OR by 2.00 per 1000/μL (Figure 2A). Patients with ST had an elevated OR of 3.26 compared to the visits of *Other*, while those with BD had an OR of 1.02 and A‐NOS a lower OR of 0.67 (Figure 2A).

Variables associated with a higher likelihood of receiving two units (OR >1) versus single RBC transfusion (OR <1) included: heart rate (OR per 10 beats/min: 1.21), fatigue (OR 2.66), dyspnea (OR 2.00), and heart disease (OR 2.13). Height increased the OR by 1.30 per 10 cm, and a prior RBC transfusion increased it by 1.74 per unit (Figure 2B). The odds of administering one unit were higher with higher Hb levels (per g/dL, OR 0.19), visits involving PLT transfusion (OR per unit 0.31), and in patients undergoing iron chelation therapy (OR 0.43) (Figure 2B).

### 3.3 Increase and course of hemoglobin levels after transfusion

**FIGURE 3:**
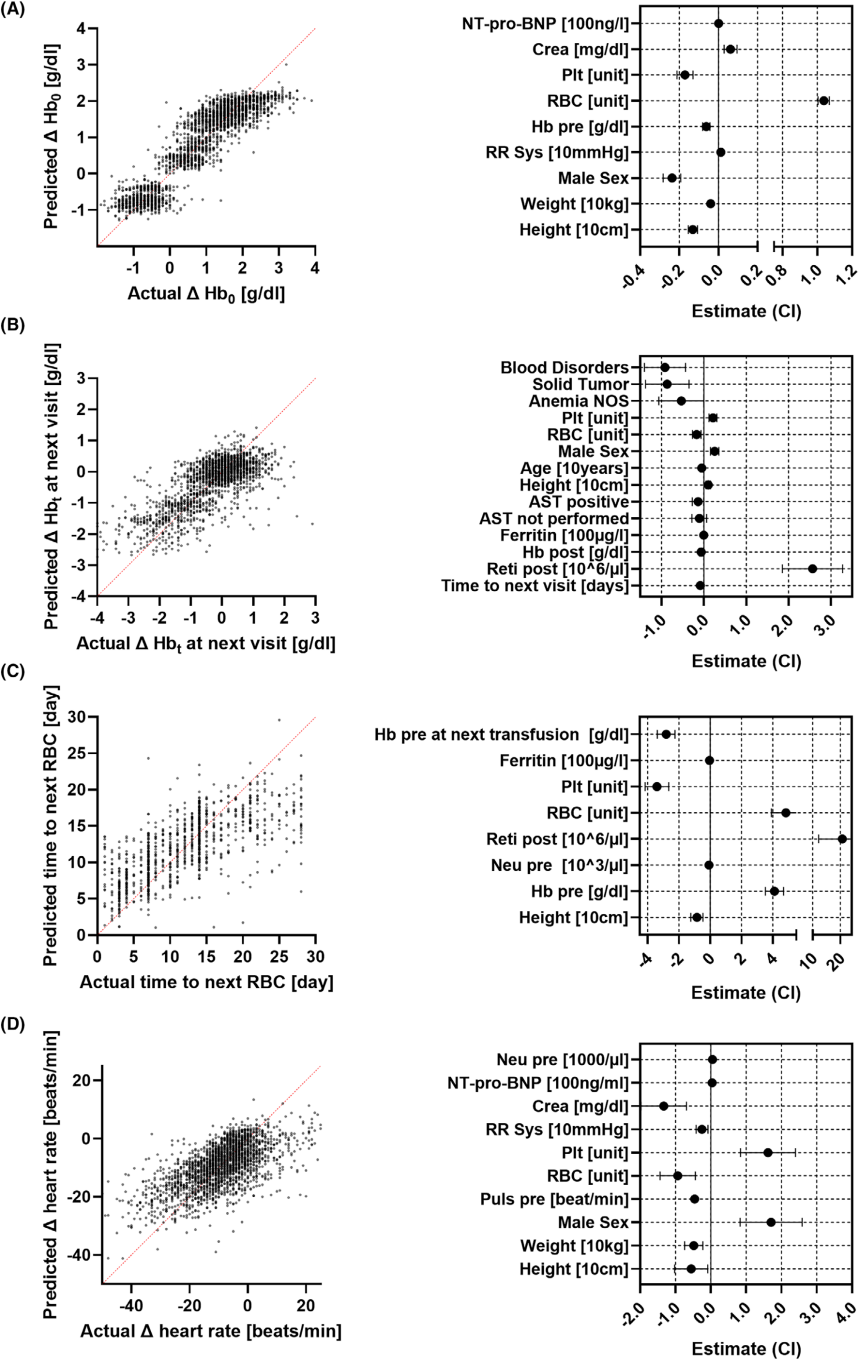
Increase of Hb levels after transfusion. Multiple linear regression models for visits in the years 2021 and 2022. Estimates are shown with 95% CI. (A) Increase of hemoglobin after transfusion (RBC or PLT), *n* = 2742 episodes could be included in this model. For the estimates for systolic blood preasure (RR sys) *p* = 0.0055 and for creatinine (Crea) *p* = 0.0002, for all other estimates *p* < 0.0001, *r* ^2^ = 0.847. (B) Change of hemoglobin at next visit; *n* = 1933 after transfusions (RBC or PLT) could be included in the model. Visits exceeding 28 days following transfusion were excluded from the analysis, as the impact of transfusion at this point is deemed to be minimal. Patients with hemoglobinopathies or gastrointestinal bleeding were also excluded. The estimates for A‐NOS (*p* = 0.051) and *AST not performed* (*p* = 0.2283) are not significant, for all other estimates is *p* ≤ 0.0499, *r* ^2^ = 0.473. (C) Time to next RBC transfusion in vistis were next transfusion was performed at a maximum of 28 days; *n* = 1063 episodes after RBC transfusions could be included in the model. Patients with hemoglobinopathies or gastrointestinal bleeding were excluded. All estimates were signifcant (*p* < 0.0001), *r* ^2^ = 0.480. (D) Change of heart frequency (Δ Puls) after transfusion of RBC or PLT or both. Visits (*n* = 2689) of all patients between 2021 and 2022 were analyzed. For all estimates *p* ≤ 0.0207, *r* ^2^ = 0.414. All *p*‐values (A–D) are listed in Tables S4–S7 and predicted hemoglobin increase derived from this linear regression in shown in Figure S10.

The increase in Hb after transfusion (ΔHb_0_) is clearly linked to the number of units transfused. In univariate analysis, one unit raised Hb by 0.6 (0.3–0.9) g/dL, while two units increased it by 1.7 (1.3–2.1) g/dL (Figure S5). Multiple linear regression revealed that ΔHb_0_ depends on the transfused units, with one RBC unit increasing Hb by 1.04 g/dL, creatinine by 0.06 g/dL/mg/dL, and minimally by NT‐pro‐BNP by 0.002 g/dL per 100 ng/L (Figure 3A). Weight (−0.04 g/dL per 10 kg), height (−0.13 g/dL per 10 cm), male sex (−0.24 g/dL), and concomitant transfusion of PLT (−0.17 g/dL per PLT unit) lowered ΔHb_0_ (Figure 3A).

Modeling Hb decline (ΔHb_t_) for patients with a visit occurring no longer than 28 days after RBC or PLT transfusion revealed the post‐transfusion reticulocytes (2.56 g/dL per 10^6^/μL) as a factor with lower ΔHb_t_, i.e., with higher Hb levels at the next visit. Factors augmenting Hb decline were height (0.10 g/dL per 10 cm), male sex (0.26 g/dL), and accompanying PLT transfusions (0.21 g/dL per unit). Factors accelerating ΔHb_t_ included the amount of RBC units (−0.17 g/dL per unit), positive AST (−0.14 g/dL), and conditions such as BD (−0.92 g/dL), ST (−0.87 g/dL), and A‐NOS (−0.53 g/dL). Daily Hb decline rates were −0.09 g/dL (Figure 3B).

Longer time to next transfusion was associated with the number of RBC units transfused (4.8 days/unit), post‐transfusion reticulocytes (20.7 days per 10^6^/μL), and pre‐transfusion Hb levels (4.1 days/g/dL), while it was shortened by PLT transfusion (−3.4 days/unit), height (−0.8 days per 10 cm), and Hb level at the subsequent transfusion (−2.8 days/g/dL) and minimally by ferritin (−0.04 days/100 μg/L) and neutrophil counts (−0.08 days per 1000/μL) (Figure 3C).

Heart rate decreased minimally after RBC transfusion at a rate of −0.9 beats/min/unit, with a higher pre‐transfusion pulse and systolic blood pressure, height, weight, and higher creatinine levels accelerating the decline. PLT transfusion (1.6 beats/min/unit), male sex, NT‐pro‐BNP, and pre‐transfusion neutrophil counts increased the post‐transfusion heart rate (Figure 3D).

### 3.4 Number of transfused RBC units and transfusion intervals

**FIGURE 4:**
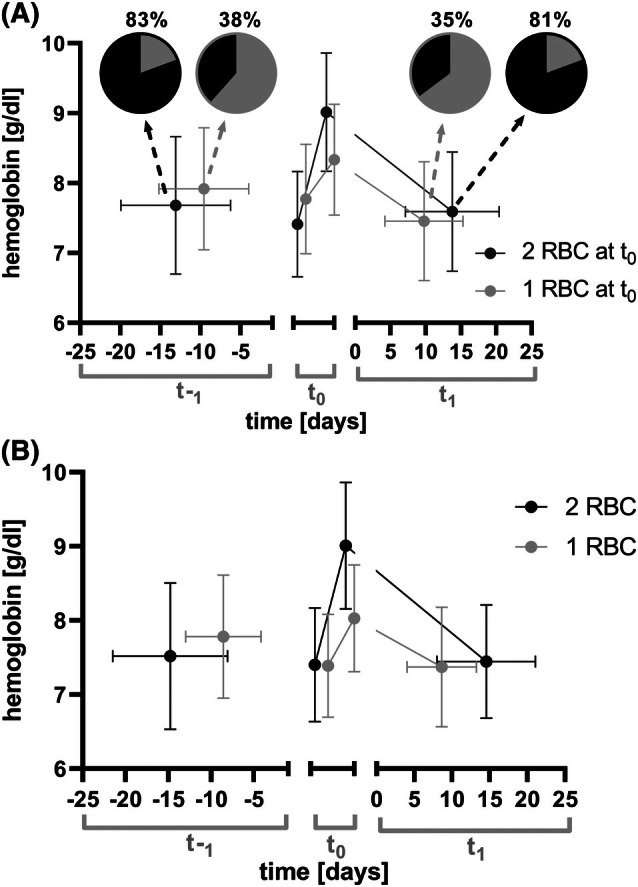
Hb course and transfusion timing in relation to the number of transfused RBC units. The course of hemoglobin (Hb) and the timing of transfusion are functions of the number of transfused red blood cell (RBC) units in patients without hemoglobinopathies or gastrointestinal bleeding who are heavily transfusion‐dependent. Data are given as mean ± SD. Transfusion episodes 2018–2022 with three RBC transfusion episodes with a maximum interval of 28 days were included. (A) Index transfusions at t_0_ were differentiated whether one (gray dots, *n* = 695) or two units (black dots, *n* = 1414) were given. These index transfusions are separated on the x‐axis for better visual discrimination. The decline of Hb to the next transfusion is shown at t_1_ with pre‐transfusion Hb levels and timing of the next transfusion. The small pie charts show the distribution of transfusion with 1 (gray) or 2 units (black), percentages above denote the part of transfusions with 2 units. (B) Index transfusions at t_0_ were identified where the same amount of units were given in the preceding and following transfusion. For continuous transfusion of one unit *n* = 345 index transfusions were analyzed and for two units *n* = 995 index transfusions. The index transfusions are separated on the x‐axis for better visual discrimination.

The models indicate a shorter interval following the transfusion of one unit compared to two units (Figures S11–S14). To further characterize these intervals and transfusion amounts, index transfusions were identified where either one or two units were given, with preceding and subsequent transfusions within 28 days. Figure 4A displays the index at t_0_, along with prior and subsequent transfusions. For transfusion of one RBC unit, the mean intervals (±SD) were −9.6 ± 5.6 days before and 9.8 ± 5.5 days after the index transfusion. For two units, the intervals were 14.0 ± 6.7 days before and 13.2 ± 7.3 days after. When one unit was transfused at t_0_, 35% of subsequent visits involved two units; when two units were given at t_0_, 81% of subsequent visits involved two units (Figure 4A).

Figure 4B shows episodes with three consecutive transfusions of one or two RBC units with transfusion intervals of no more than 28 days. The difference in intervals (one versus two units) increased: for one unit (*n* = 345), the intervals were 8.6 ± 4.4 and 8.6 ± 4.6 days; for two units, they were 14.8 ± 6.7 and 15.6 ± 6.5 days (*n* = 995) (Kruskal–Wallis *p* < .0001).

A single unit transfusion strategy has been shown to save 25 to 29% of RBC in hemato‐oncologic patients.^14^, ^15^ From the linear regression model (Figure 3C), we calculated the effect of a one versus two RBC unit strategy for 1 year (Figures S10–S14). Depending on sex, pre‐transfusion Hb level, concomitant transfusion of PLTs, height, and weight, the calculated RBC reduction ranged from 33% to an 18% increase in RBC demand for a single unit strategy compared to a two unit strategy (Figures S13 and S14). Considering the intervals derived from Figure 4A, the saving of RBC units by a strict one‐unit strategy revealed 30% and from Figure 4B, 17%. Analyzing a series of transfusions consisting of at least three consecutive visits with equal numbers of units, heavily transfusion‐dependent patients who received either single‐unit or two‐unit transfusions switched their transfusion strategy after a median of 41 days and 7 visits in the single‐unit group and after 91 days and 7 visits in the two‐unit group, suggesting no potential for red blood cell (RBC) savings (Figure S8). Estimates from the linear regression model (Figure 3C, Figure S14) and transfusion intervals (Figure 4B) revealed that the one‐unit strategy was associated with a 30%–57% (71%) increase in visit rate.

### 3.5 Hemovigilance

Between 2021 and 2022, 14 adverse transfusion reactions were recorded in the hemovigilance system for the study cohort. In eight cases, RBC units were transfused; one involved only RBC units, and in seven cases, PLT units were also transfused. All reactions were classified as febrile and non‐severe. Five patients were affected, with two experiencing reactions two or three times. Both patients with multiple reactions were female (MDS and A‐NOS). The other three included a female with urothelial carcinoma and two males with AML. In cases involving both PLT and RBC units, the causative product could not be identified (Table S3).

## 4 DISCUSSION

Nearly 75% of patients in the outpatient clinic have BD or ST (Table 1), which accounts for 88% of visits. Among BD patients, those with AML, MPN, MDS, and AA/PNH received 49% of RBC units, despite representing only 36% of patients, and often also required PLT transfusions due to thrombocytopenia. While some publications have recently been published for MDS,^1^, ^17^, ^20^ MPN or AML were rarely addressed as candidates for outpatient RBC transfusion.

For ST patients, RBC transfusion demand was associated with the underlying disorder (Figure S1). The median age of 70 years and slight male predominance in BD and ST groups matches regional epidemiological data.^21^ Some A‐NOS patients had bi‐ or tricytopenia, possibly due to undiagnosed MDS, as further investigation was declined.

The median pre‐transfusion Hb concentration in our study was 7.5 g/dL and thus in accordance with a widely accepted transfusion trigger of 7–8 g/dL.^6^, ^7^, ^12^ Transfusion triggers in the outpatient setting are best investigated for MDS patients,^1^, ^2^, ^17^, ^22^ but the optimal trigger is still under debate. Our study revealed a substantial inter‐patient and intra‐patient variability of pre‐transfusion Hb levels (Figure 1, Figures S3 and S4) reflecting the variability of RBC transfusion in the outpatient setting. Differences in median pre‐transfusion Hb levels across age groups reached up to 0.4 g/dL in the groups of <55 compared to >75 years old. We consider this difference to be clinically meaningful. Higher pre‐transfusion Hb levels in older patients correlated with increased odds of transfusion per visit (Figure 2A), suggesting a clinical need for higher Hb levels in older individuals. As suggested by others^23^ transfusion triggers should be carefully adjusted to the situation of the patient. Prospective studies must clarify whether this variance is the expression of different patient needs at a specific point in time or the need for optimization of transfusion strategies in general.

We assumed that conditions like fatigue, dyspnea, and heart disease would be significantly associated with higher pre‐transfusion Hb levels (Figure S2). Although a statistically significant correlation was confirmed, the absolute differences in the pre‐transfusion Hb levels between patients with and without these conditions were very small. However, these conditions significantly increased the odds of transfusion and receiving two units (Figure 2A,B), indicating their importance in transfusion decision‐making. Other statistically significant factors included higher pulse rate, age, and PLT count. The documentation of symptoms in the patient records might be incomplete due to the documentation of only exceptional symptoms leading to underreporting and underestimation of minor symptoms in our analysis.

ST patients had higher odds for RBC transfusion at a visit compared to BD and A‐NOS. For diagnostic groups, the visit practice has to be taken into account as BD patients might get more controls of blood cell counts and observe visits where only platelet transfusions are given. The most significant predictor for an RBC transfusion at a visit was the pre‐transfusion Hb, with higher levels showing reduced odds. The effort of traveling to the transfusion center was previously anticipated.^22^ Contrary to expectations, travel distance was associated with slightly lower odds, suggesting no liberal transfusion trend for those with a longer travel. The effect of PLT count may be a confounder due to the practice of transfusing PLTs but not RBCs in thrombocytopenic patients.

When choosing whether to transfuse one or two units of RBC, symptoms, heart disease, and higher pulse favored two units, while higher Hb levels, concomitant PLT transfusion, and iron chelation favored one unit (Figure 2B). Height and prior transfusions increased the likelihood of two units. This is in line with the negative effects of these measures on ΔHb_0_ (Figure 3A).

Two RBC units increased Hb by a factor of 2.8 compared to one unit (Figure S5), possibly due to redistribution effects post‐transfusion as sampling occurred immediately after the end of RBC transfusion and therefore the time since the transfusion was started was longer when two RBC units were transfused than when only one RBC unit was transfused. However, our ΔHb_0_ results are in line with Reikvam et al.^24^ who correlated Hb increase with the total Hb transfused per BV (Figure S9) and determined Hb 15 minutes after equilibration. Co‐administration of PLT, pre‐transfusion Hb, and factors that might interfere with blood volume, such as creatinine, systolic blood pressure, NT‐pro‐BNP, sex, height, and weight were additional influencing factors of ΔHb_0_.

The Hb decline post‐transfusion (ΔHb_t_) was −0.09 g/dL per day. Stabilizing factors included post‐transfusion reticulocytes, height, and male sex. The amount of RBC units is inversely correlated to ΔHb_t_. This can be explained by the fact that the decline reflects the non‐reproduction of transfused Hb over time. The more allogeneic Hb that was transfused, the more Hb was prone to decline, which leads to major changes in Hb levels over time. It was speculated that adaptation to lower Hb levels is better when changes remain low.^22^ Higher ferritin levels and diagnostic categories could be indicators of an increased risk of anemia, i.e., a more severe erythropoietic failure requiring more transfusions and thus having an influence in the linear regression models for Hb decline or time to the next transfusion (Figure 3B,C).

The analysis of the decline in heart rate post‐transfusion was included post hoc as it might be considered a surrogate for the clinical success of RBC transfusion. The analysis demonstrates that PLT and RBC transfusions exerted distinct effects, suggesting that fluid administration alone does not fully account for the changes. Higher pre‐transfusion pulse rates showed a greater response to RBC transfusions, while weight and height were associated with reductions in pulse rates; male sex was linked to an increase, which may be attributed to the absence of a strict protocol for determining pulse rate (e.g. sitting for at least 15 min) and other factors that could mask the decreasing effect of transfusion. Therefore, the results of this model should be interpreted as indicative of a trend but should be further considered as an end point in future studies.

Our estimates of the RBC‐saving potential of a single‐unit strategy, derived from various models (Figures 3C and 4B, Figure S8), differ from previously published results,^14^, ^15^ particularly in specific clinical scenarios where no savings of RBC use were observed. Although noteworthy, these studies did not address patient characteristics that we identified, suggesting differential effects for a single‐unit strategy. The Hb increments after transfusion also vary substantially with height, sex, pre‐transfusion Hb levels, and co‐administration of PLT (Figure 2A, Figure S10). The potential savings in RBC units transfused are offset by a higher number of visits, which, in the case of comorbid patients in need of transfusion, can in turn result in additional burden and costs. These aspects must be carefully balanced against each other. It is possible to substitute the number of transfused units (Figure 3C) with the post‐transfusion hemoglobin (Hb) level in the linear regression model for time to next transfusion (Figure S7), emphasizing that transfusion decision‐making should focus on the target Hb achievable in a given patient rather than on the number of units transfused. Overall, our data do not support a strict one‐unit policy for unselected heavily transfused outpatients, as suggested by others for inpatients.^13^ Thus, it is critical for future studies to exactly consider dose and thus focus on a hemoglobin target as an important variable or even endpoint.

The hemovigilance data indicated no significant adverse transfusion reactions, with a rate of ≤0.24% per transfusion visit, affecting ≤0.68% of outpatients, underlying the good safety profile of this procedure.

The study's retrospective nature and reliance on patient records without predefined systematic assessments are significant limitations of our study as documentation of symptoms was inconsistent. However, the large patient number and documented visits provide a comprehensive overview of real‐world outpatient transfusion patterns in patients with various diagnoses for which there is currently a substantial knowledge gap.

Outpatient transfusion is primarily relevant to hemato‐oncology patients who may also need PLT transfusions. Post‐transfusion Hb trajectories vary, requiring individualized dose (in terms of units) and interval adjustments to optimize outcomes. Our findings provide valuable insights for designing clinical trial protocols for outpatient RBC transfusion. There have been many trials on Hb triggers but not on Hb targets that depend on age and gender differences, co‐administered blood products, body measurements, and diagnoses as important clinical determinants. This adds some complexity to decision‐making in RBC transfusion that has to be taken into account in the designs of future prospective clinical trials. A one‐size‐fits‐all approach in terms of triggers and doses might not meet the requirements of patients that need long‐term transfusion support.

## AUTHOR CONTRIBUTIONS

SK designed the study. DF, NH, BR, SK performed statistics. DF, HS, NH, BR, SK analyzed and interpreted data. SK, NH, and HS wrote the manuscript. CW performed antibody testing. BJ, CW provided hemovigilance data. BJ, SK, BH, AMH, CK, HS treated patients. All authors have approved the manuscript.

## CONFLICT OF INTEREST STATEMENT

This work is part of the doctoral thesis of NH. All other authors are employed by the Blood Transfusion Service Baden‐Württemberg‐Hessen or its affiliates which provided the blood products that were transfused within the study.

## ETHICAL APPROVAL

Ethics committee University Hospital Ulm, Number 205/23.

## Supporting information


**Data S1.** Supporting Information.

## Data Availability

Anonymised data will be available upon request for independent review panel‐approved research proposals with a signed data sharing agreement. The data will be available after approval and data sharing agree ment is in place until 3 years after publication.
