# Supplementary material for: Patient characteristics and clinical determinants of outpatient red cell transfusions
Source: Transfusion. 2025 Oct 15;65(11):2065–76. doi: 10.1111/trf.18428 (PMC12618906; doi:10.1111/trf.18428)
Supplement: Supplementary file 1 — Data S1. Supporting Information. [file TRF-65-2065-s001.pdf]

Supplement

Patient Characteristics and Clinical Determinants of Outpatient Red Cell Transfusions

Nora Hemesath, Daniel Fürst, Marianne Holl, Bianca Ries, Astrid Marx-Hofmann, Christine Kroll, Britta Höchsmann, Bernd Jahrsdörfer, Christof Weinstock, Hubert Schrezenmeier, Sixten Körper[1]

Institute for Clinical Transfusion Medicine and Immunogenetics Ulm, German Red Cross Blood Transfusion Service Baden-Württemberg-Hessen and University Hospital Ulm and Institute of Transfusion Medicine, University of Ulm.

[1] Correspondence: Dr.med.Sixten Körper, Institute for Clinical Transfusion Medicine and Immunogenetics Ulm, German Red Cross Blood Transfusion Service Baden-Württemberg-Hessen and University Hospital Ulm and Institute of Transfusion Medicine, University of Ulm, e-mail: *s.koerper@blutspende.de*

Content

Suppl. fig. 1. Patients that were transfused in the years 2018-2022. Visits and transfused RBC units in the years 2021 or 2022 are shown. 3

Suppl. Fig. 2. Pre-transfusion Hb levels by sex and condition without hemoglobinopathias and GI-bleeding. 4

Suppl. fig. 3. Pre-transfusion Hb level by patients with n≥10 transfusions (n=97) (A-B). 5

Suppl. Fig. 4. Pre-transfusion Hb-levels at visits from patients with MDS, MPN, AML and AA/PNH. 6

Suppl. Fig. 5. Change of hemoglobin and reticulocytes after transfusion stratified by number of transfused RBC units. 6

Table 1: Visits 2018 – 2022 7

Table 2: Visits 2018-2020 9

Table 3: transfusion reactions 2021 / 2022 10

Suppl. Fig. 6. Quality Parameters of odds ratio (figure 2). 11

Suppl. fig. 7. Alternative model for figure 3 C: time to next transfusion 12

Suppl. fig. 8. Duration of transfusion episodes with an equal amount of RBC units. 13

Suppl. fig. 9. Increase in Hb [g/dl] after transfusion versus transfused hemoglobin [g] per BV. 14

Suppl. Fig. 10. Predicted Hb increase after RBC transfusion derived from the linear regression model for the increase of Hb after transfusion (figure 3A). 16

Suppl. Fig. 11 Transfused RBC units (A) and visits per year (B) by height and different pre-transfusion Hb levels without concomitant administration of PLTs. 17

Suppl. fig. 12. Transfused RBC units (A) and visits per year (B), with concomitant transfusion of PLTs . . 18

Suppl. fig. 13. Ratios of transfused units per years of a two unit versus a one unit strategy for different pre-transfusion Hb levels without PLT (A) and with PLT co-administration (B). 19

Suppl. fig. 14. Ratios of transfusion visits per year of a two unit versus a one unit strategy for different pre-transfusion Hb levels without PLT (A) and with PLT co-administration (B). 20

Supplemental Methods 21

Supplemental Table 4: ΔHb$_0$ 23

Supplemental table 5: ΔHb$_t$ next visit 24

Supplemental table 6: Time to next RBC 25

Supplemental table 7: Δ heart rate 26

Literature 26

Note: Supplemental figure 8 and 10 to 14: Data analysis assistance was provided using ChatGPT 4.0, an AI language model developed by OpenAI, for generating visualizations of results

Supplemental figure 1

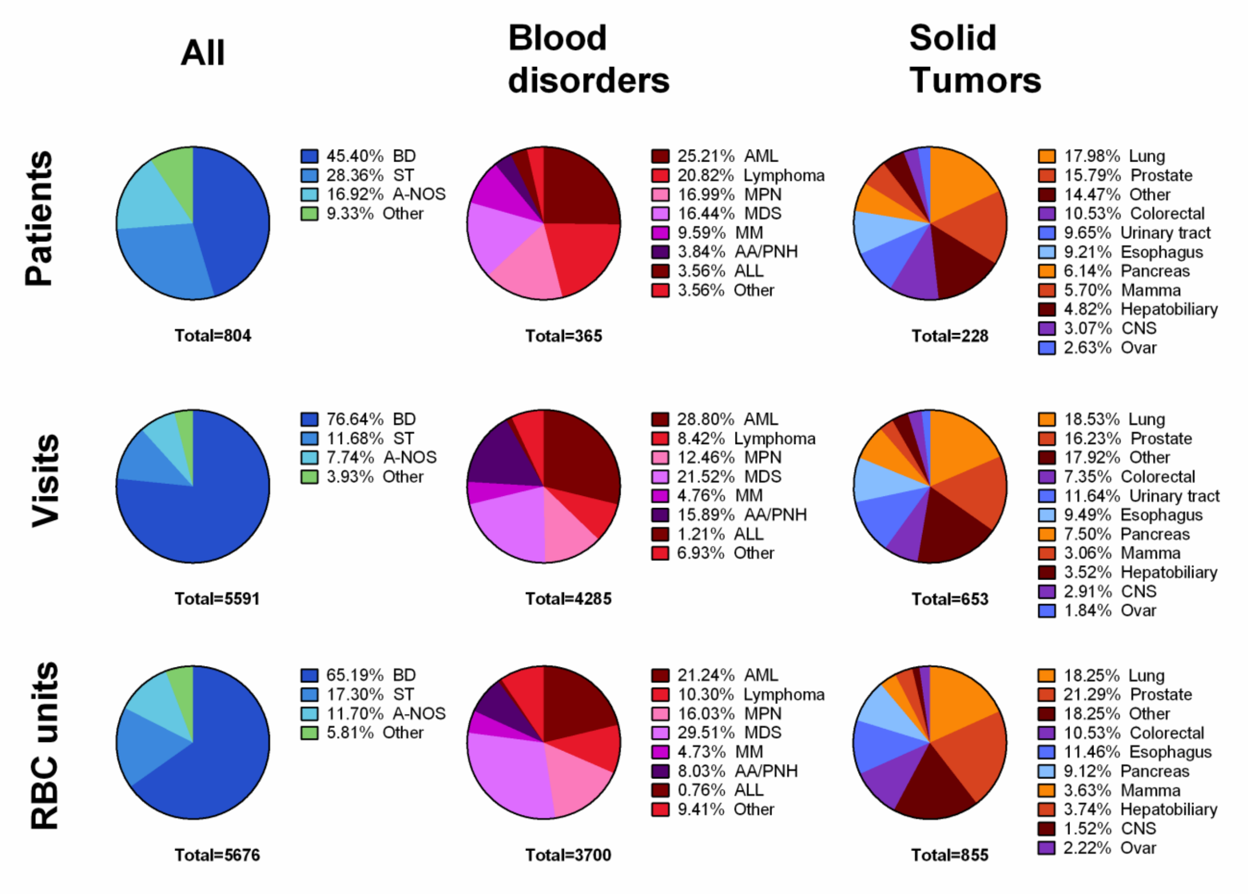

Suppl. fig. 1. Patients that were transfused in the years 2018-2022. Visits and transfused RBC units in the years 2021 or 2022 are shown.

Proportion of patients with blood disorders (BD), solid tumours (ST) anaemia not otherwise specified (A-NOS) and *Others* with their share on visits and transfused RBC units.

Supplemental figure 2

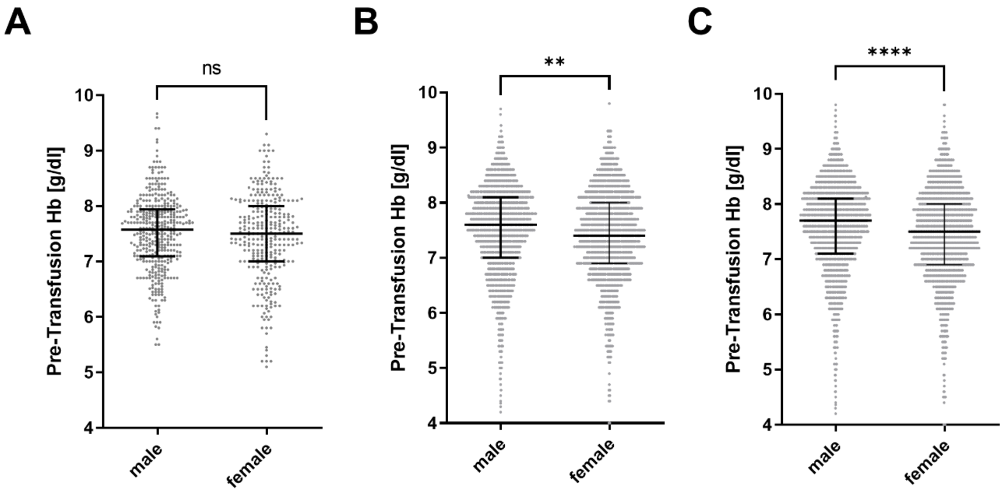

	n=390	n=310
**Mean**	7.51	7.43
**Std. Deviation**	0.69	0.79

n=1751	n=1213
7.48	7.40
0.84	0.85

n=2474	n=1701
7.58	7.42
0.82	0.86

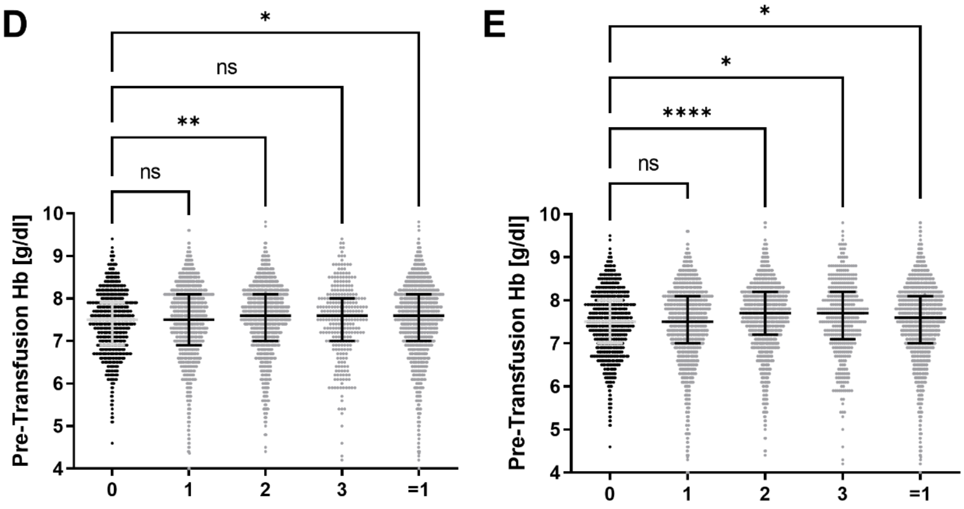

	n=795	n=1076	n=806	n=287	n=2169
**Mean**	7.41	7.42	7.52	7.46	7.46
**Std. Deviation**	0.73	0.90	0.84	0.90	0.88

n=1019	n=1585	n=1180	n=391	n=3156
7.48	7.45	7.60	7.59	7.53
0.74	0.90	0.80	0.91	0.87

Suppl. Fig. 2. Pre-transfusion Hb levels by sex and condition without hemoglobinopathias and GI-bleeding.

(A) Pre-transfusion Hb levels of individual patients 2018 to 2022. (B) Visits occuring 2021 or 2022. (C) Visits occuring 2018 to 2022. (A-C) Mann-Withney-Test was performed for testing significance. (D-E) Pre-transfusion Hb levels stratified by clinical condition and symptoms: patients with no symptoms or heart disease, denoted as “0”. Symptoms like fatigue or dyspnea or the accompanying heart disease were counted as a condition. Numbers give the amount of conditions observed at a visit. (D) Visits occurred in 2021 or 2022. (E) Visits occurred between 2018 to 2022. Groups were compared by Kruskal-Wallis-Test.

Supplemental figure 3

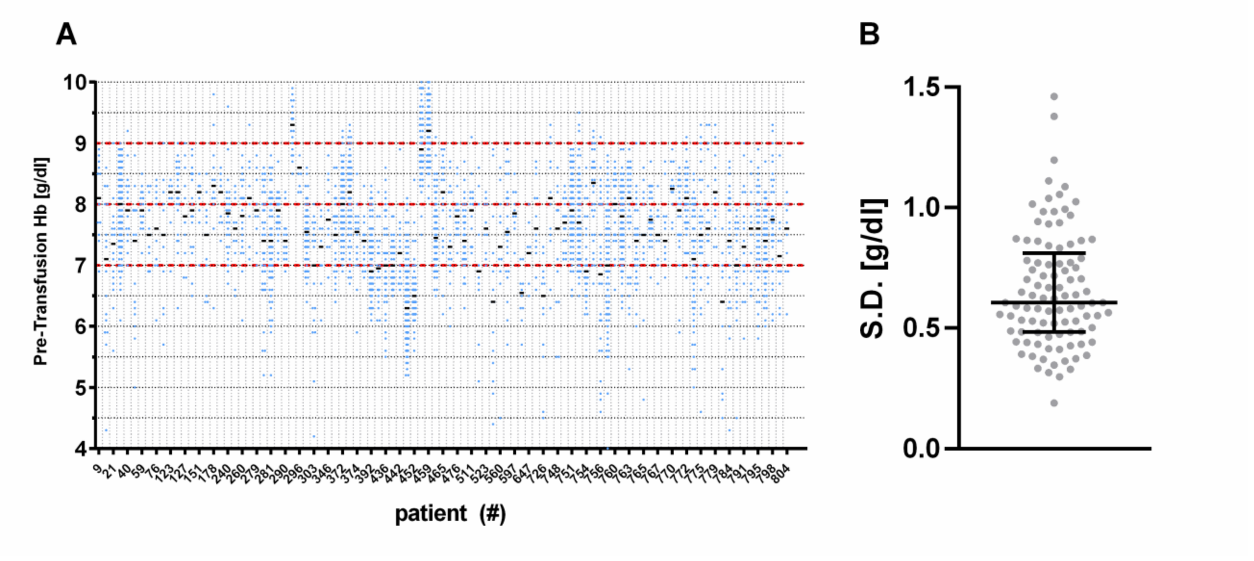

Suppl. fig. 3. Pre-transfusion Hb level by patients with n≥10 transfusions (n=97) (A-B).
(A) Pre-transfusion Hb levels at visits of individual patients (blue dots). For better reading only every second patient number is displayed. Small black lines denote the mean of pre-transfusion Hbs. (B) shows the standard deviations of the individual pre-transfusion Hbs (grey dots) shown in A with a median of 0.61 and interquartile ranges (0.48; 0.81) g/dl indicating a substantial variance.

Supplemental figure 4

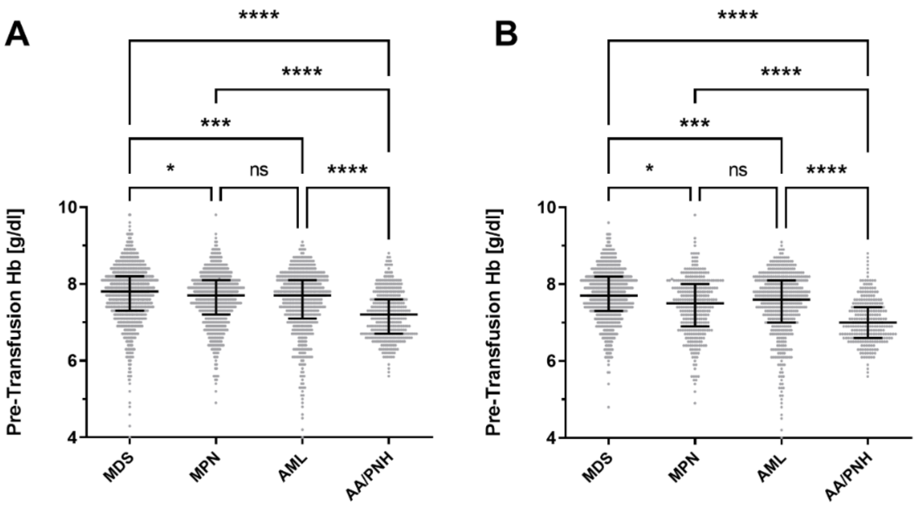

	n=1107	n=573	n=516	n=357
Mean	7.71	7.59	7.48	7.19
Std. Deviation	0.75	0.72	0.90	0.62

n=619	n=340	n=468	n=264
7.71	7.43	7.46	7.05
0.69	0.75	0.91	0.58

Suppl. Fig. 4. Pre-transfusion Hb-levels at visits from patients with MDS, MPN, AML and AA/PNH. (A) Visits occurring in 2018-2022. (B) Visits occurring in 2021 or 2022. Kruskal-Wallis-Test was used to determine statistical significance.

Supplemental figure 5

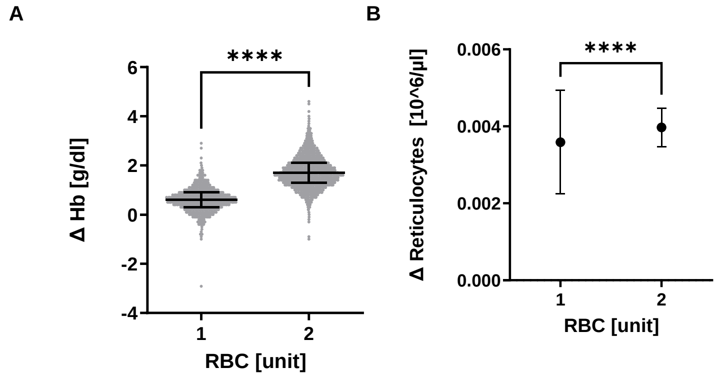

	n=1112	n=3590
Median	0.6	1.7
25% Percentile	0.3	1.3
75% Percentile	0.9	2.1
Mean	0.60	1.72
Std. Deviation	0.48	0.63

	n=1056	n=3352
Median	0	0.01
25% Percentile	0	0
75% Percentile	0.01	0.01
Mean [10^3/µl]	3.589	3.971
Std. Deviation	0,04363	0,02888

Suppl. Fig. 5. Change of hemoglobin and reticulocytes after transfusion stratified by number of transfused RBC units.

Transfusion episodes of all patients from 2018 to 2022 were analyzed. (A) Change of hemoglobin. Two units increased the hemoglobin 2.8 times more compared to one unit. (B) The increase in reticulocytes was only 1.1 times more pronounced when two units were transfused.

Table 1: Visits 2018 – 2022

	Total N=804 (100%)	BD N=365 (46%)	Solid Tumor N=228 (28%)	A-NOS N=137 (17%)	Other N=74 (9%)	BD vs. ST	A-NOS vs. BD	A-NOS vs. ST
Demographic and clinical characteristics								
Gender, no (%)						-	-	-
Female	350 (44)	143 (39)	93 (41)	81 (60)	33 (44)			
Male	454 (56)	222 (61)	135 (59)	55 (40)	42 (56)			
Median age, years (IQR)	72.6 (61.6; 81.7)	69.6 (58.5; 78.9)	69.9 (61.5; 78.5)	82.1 (73.8; 87.8)	81.5 (70.6; 88.14)	ns	****	****
Heart disease, no (%)	248 (30.8)	87 (23.8)	53 (23.2)	70 (51.5)	38 (50.7)	-	-	-
Only RBC transfusion, no (%)	560 (70)	171 (47)	192 (84)	129 (94)	68 (92)	-	-	-
Only with PLT transfusion, no (%)	50 (6)	42 (12)	5 (2)	1 (0.7)	2 (3)	-	-	
Patients with PLT and RBC transfusion, no (%)	180 (22)	142 (39)	29 (13)	7 (5)	2 (3)	-	-	-
FFP, no	2	2	0	0	0	-	-	-
No transfusion, no	14	10	2	0	2	-	-	-
Traveldistance, km (IQR)	17.0 (10.0; 32.0)	18.0 (10.0; 45.0)	18.0 (10.3; 32.8)	14.0 (8.0; 21.0)	18.0 (14.0; 24.0)	ns	**	*
Patients with visits before 2021, no	164	98	28	22	16	-	-	-
**Visits**	7824	6154	762	615	293	-	-	-
Time since diagnosis at visit, month (IQR)	23.4 (7.8; 77.4)	24.8 (8.7; 85.7)	27.5 (7.6; 64.4)	7.7 (1.1; 23.0)	4.4 (2.6; 13.5)	-	-	-
Symptoms, no (%)	3420 (44)	2325 (38)	527 (69)	397 (65)	171 (58)	****	***	ns
Dyspnea	1458 (19)	925 (15)	227 (30)	220 (36)	86 (29)	-	-	-
Fatigue	3169 (41)	2157 (35)	495 (65)	360 (59)	157 (54)	-	-	-

Visits with RBC transfusion, no	4748	3407	625	464	252	-	-	-
Visits with 1 RBC transfusion	1129	946	61	64	58			
Visits with 2 RBC transfusion	3618	2461	564	399	194			
Visits with 3 RBC transfusion	1	0	0	1	0			
RBC transfusion, units	8368	5868	1189	865	446	****	****	ns
Per patient ever RBC transfused, median (IQR)	4.0 (2.0; 9.0)	5.0 (2.0; 17.0)	3.0 (2.0, 6.0)	2.0 (2.0; 7.0)	3.5 (2.0 ; 5.0)	****	****	ns
Plt transfusion, units	2982	2792	104	81	5	ns	ns	ns
Per patient ever Plt transfused, median (IQR)	3.0 (1.0; 9.0)	4.0 (1.0; 10.8)	1.0 (1.0; 3.0)	12.0 (2.0; 43.0)	1.0 (1.0; 2.5)	**	ns	*
AKS pos., no (%)	61 (8.3)	39 (12.5)	11 (5.0)	9 (6.7)	2 (3.0)	-	-	-
Neutrophile count, 10^3/µl (IQR)	3.9 (2.1; 5.7)	2.7 (1.3; 5.1)	4.3 (3.0; 6.4)	4.4 (2.9; 5.6)	4.5 (3.2; 6.1)	****	****	ns
Hemoglobin, g/dl (IQR)	7.5 (7.0; 8.0)	7.6 (7.1; 8.0)	7.7 (7.2; 8.0)	7.2 (6.6; 7.8)	7.6 (7.0; 8.2)	ns	**	***
Reticulocyte count, 10^6/µl (IQR)	0.059 (0.037; 0.090)	0.047 (0.025; 0.080)	0.064 (0.042; 0.094)	0.066 (0.050; 0.088)	0.073 (0.050; 0.101)	****	****	ns
Plt, 10^3/µl (IQR)	166.0 (57.3; 287.4)	69.3 (26.0; 179.3)	221.0 (114.5; 310.3)	262.0 (168.0; 351.0)	213.8 (146.0; 308.5)	****	****	ns
GFR, ml/min (IQR)	65.0 (44.1; 89.3)	67.6 (49.1; 91.0)	76.1 (51.1; 102.2)	54.6 (35.3; 76.2)	40.8 (24.1; 64.2)	ns	***	****
Ferritin, µg/l (IQR)	543.5 (85.6; 1523.0)	1048.0 (430.8; 2930.0)	541.7 (173.7; 1472.0)	39.1 (13.5; 343.7)	83.6 (41.5; 277.1)	****	****	****
Pro-NT-BNP, ng/l (IQR)	807.4 (326.6; 2218.0)	832.1 (299.3; 2206.0)	622.3 (287.9; 1383.0)	1121.0 (388.5; 2845.0)	2690.0 (319.7; 14970.0)	ns	ns	*

Table 2: Visits 2018-2020

	Total	BD	Solid Tumor	A-NOS	Other
**Demographic and clinical characteristics**					
Patients	164	98	28	22	16
Visits	2233	1869	109	182	73
Diagnosis		AA/PNH; ALL; AML; MPN; HB; Lymphoma; MDS; MM; TTP	Bronchial; Ca; Colorectal; Esophagus; Mamma; Pancreas; Prostate		GI-Bleeding; Renal insufficiency
Gender, no (%)					
Female	77 (47)	46 (46)	11 (39)	14 (64)	6 (38)
Male	87 (53)	52 (54)	17 (61)	8 (36)	10 (62)
Median Age, years (IQR)	75.8 (66.7; 81.0)	73.6 (66.1; 79.9)	80.1 (69.6; 87.3)	86.1 (85.7; 89.1)	82.0 (80.8; 88.5)
Visits with RBC Transfusion, no	1471	1191	106	109	65
1 RBC	250	214	5	17	14
2 RBC	1221	977	101	92	51
RBC Transfusions, units	2692	2168	207	201	116
PLT Transfusions, units	558	506	0	51	0
Hb pre, g/dl (IQR)	8.2 (7.4; 9.0)	8.2 (7.5; 9.0)	7.7 (6.9; 8.2)	8.6 (7.6; 9.5)	7.8 (7.2; 8.6)
Ferritin, µg/l (IQR)	1124.0 (398.1; 2558.0)	1332.0 (493.9; 2858.0)	506.9 (75.7; 1155.0)	557.2 (31.9; 1535.0)	55.2 (40.0; 92.5)

Table 3: transfusion reactions 2021 / 2022

Patient number	Reaction number	Age [years]	Sex	Diagnosis	Transfused units	Description	Classification	grading
761	1	59	w	Urothelial Ca	2 RBC<br>2 PLT	fever	Febrile TR	mild
761	-	59	w	Urothelial Ca	2 PLT	Malaise, fever <39°C	Febrile TR	moderate
766	1	63	m	AML	1 RBC<br>1 PLT	Fever <39°C	Febrile TR	mild
683	1	81	m	AML	2 RBC<br>1 PLT	fever	Febrile TR	mild
796	1	73	m	MDS	1 RBC<br>1 PLT	Body temperature <39°C	Febrile TR	mild
796	2	73	m	MDS	1 RBC	Malaise, chillls	Febrile TR	moderate
724	1	86	w	A-NOS	1RBC<br>2 PLT	Fever <39°C	Febrile TR	mild
724	2	86	w	A-NOS	1 RBC<br>1 PLT	Fever <39°C, chills	Febrile TR	mild
724	3	86	w	A-NOS	1 RBC<br>1 PLT	Malaise, nausea, chills,<br>Fever <39°C	Febrile TR	mild

Supplemental figure 6

A

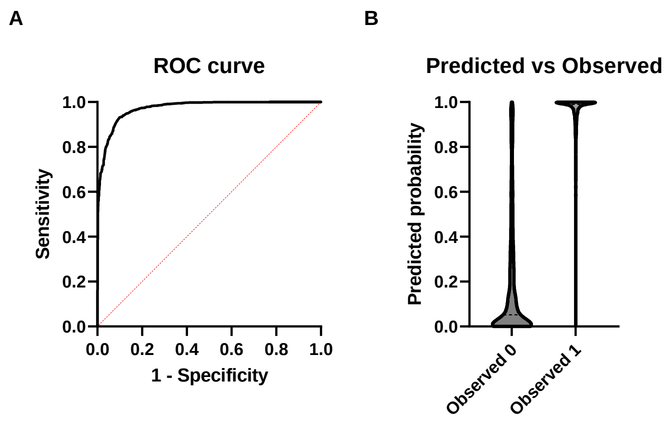

B

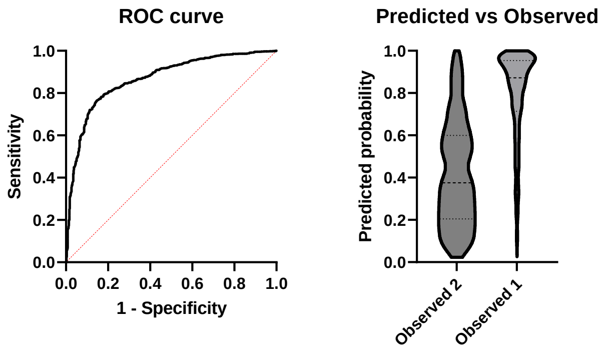

Suppl. Fig. 6. Quality Parameters of odds ratio (figure 2).

A: Figure refers to Fig. 2 A in the main manuscript. Negative predictive power: 88.9 %, positive predictive power: 93.7%. Tjur´s R squared 0.730. B: figure refers to figure 2B in the main manuscript. Negative predictive power 70.9% and positive predictive power 82.5%. Tjur´s R squared 0.377.

Supplemental figure 7

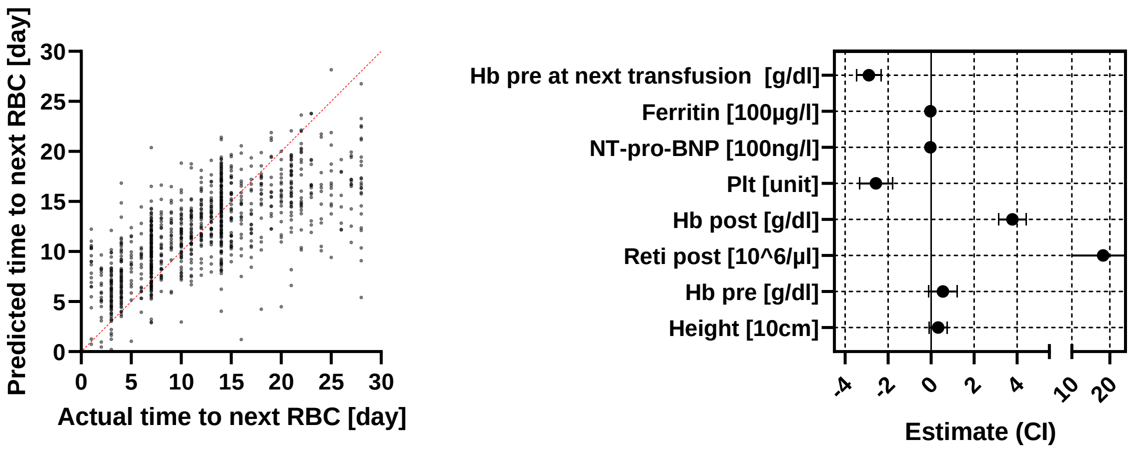

Suppl. fig. 7. Alternative model for figure 3 C: time to next transfusion.
Transfused RBC units were substituted by Hb after transfusion (*Hb post*), r^2=0.486, n= 1069. The comparable result to figure 3 C emphasizes the relevance of target Hb. P-values are summarized in supplemental table 5, column 9.

Supplemental Figure 8

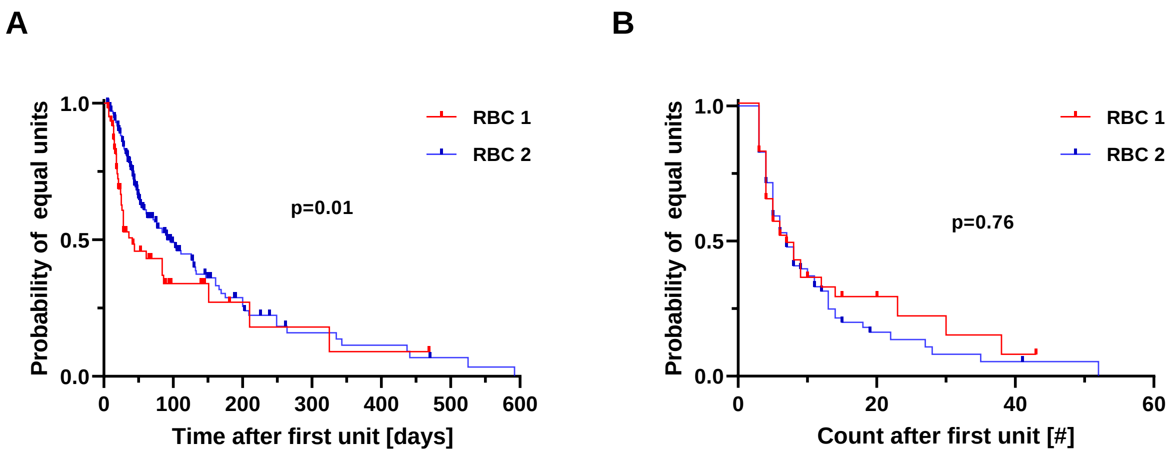

Suppl. fig. 8. Duration of transfusion episodes with an equal amount of RBC units.
Transfusion visits with an interval ≤ 28 days and an equal amount of units in a sequence of least 3 units were included in the analysis. Kaplan-Meier-Statistics were used to analyze the duration of episodes with transfusion of constantly one or two units of RBC, a change in the amount was counted as an event. Data were censored when the interval extended 28 days or when no consecutive RBC transfusion was given. Episodes and calculation of intervals was done by ChatGPT 4.0 in Excel. Patients with gastrointestinal bleeding or hemoglobinopathies where excluded. In the one unit group (red line) a total of n=62 episodes of n=35 patients with a total of n=469 visits could be included in the analysis. For the two unit group (blue line) n= 246 episodes of n=126 patients with a total of n=1490 visits are included. (A) Duration (days) of equal unit transfusion episodes. The median of switched strategy was reached after 41 days in the one unit group and 91 days in the two unit group. (B) Count of visits of equal unit transfusion visits. The median of switched strategy was reached after the seventh visit in both groups.

The curves for the transfusion visits show no difference between groups. Since the median for both groups is reached at the seventh transfusion visit (B), the two-unit group should have received twice as many units as the one-unit group. However, because the one-unit group reached the median earlier, at day 41, compared to day 91 for the two-unit group (A), our data do not support an advantage in terms of transfused units when considering the need for units over time.

Supplemental Figure 9

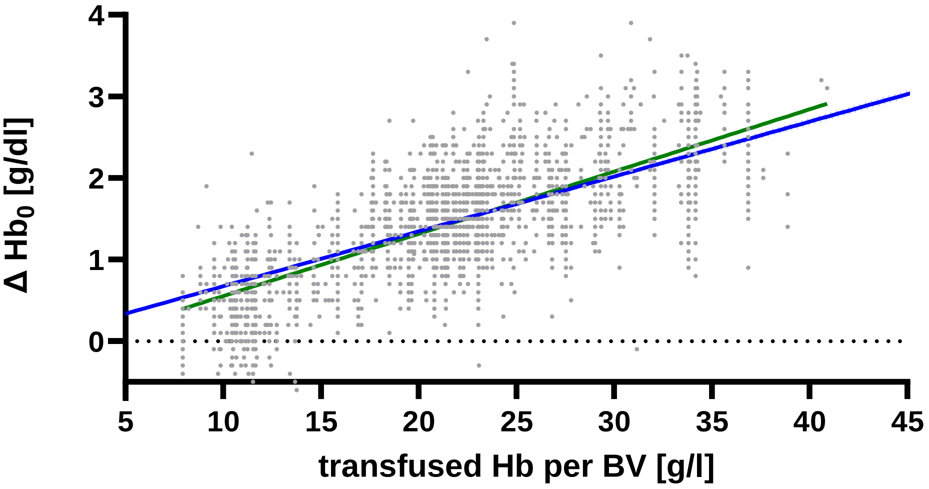

Suppl. fig. 9. Increase in Hb [g/dl] after transfusion versus transfused hemoglobin [g] per BV. Measurements of Hb content for each unit were not available. Hb content per blood volume were estimated with the assumption that one unit contains 55.6 g (derived from quality control data of the years 2021 and 2022). N=3010 transfusions with available BV. Blood sampling was performed immediately after transfusion. The regression fitted through the origin (x=0 and y=0) (green line) and has the following equation: y= 0.06725x + 0.0 ($p<0.0001$). The regression fitted without restrictions (blue line) has the following equation: y = 0.07630x – 0.2110, with r^2=0.54 and $p< 0.0001$. Reikvam et al. (1) published results on ΔHb_0 with blood sampling 15 minutes after the end of transfusion to let the Hb equilibrate. Graphical estimation from revealed an equation of y = 0.0687x +0.0. These results of ΔHb_0 are comparable to ours despite the difference in sampling time.

Supplemental figure 10

A

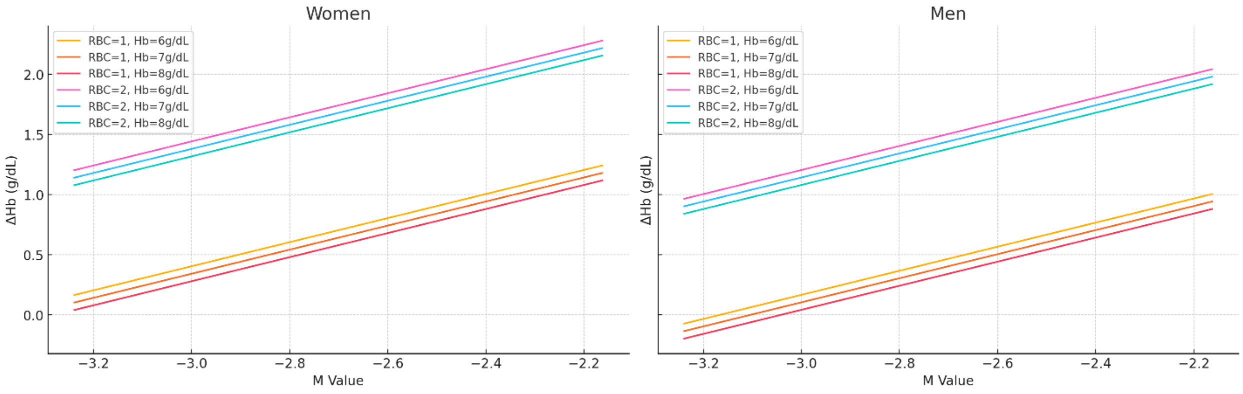

B

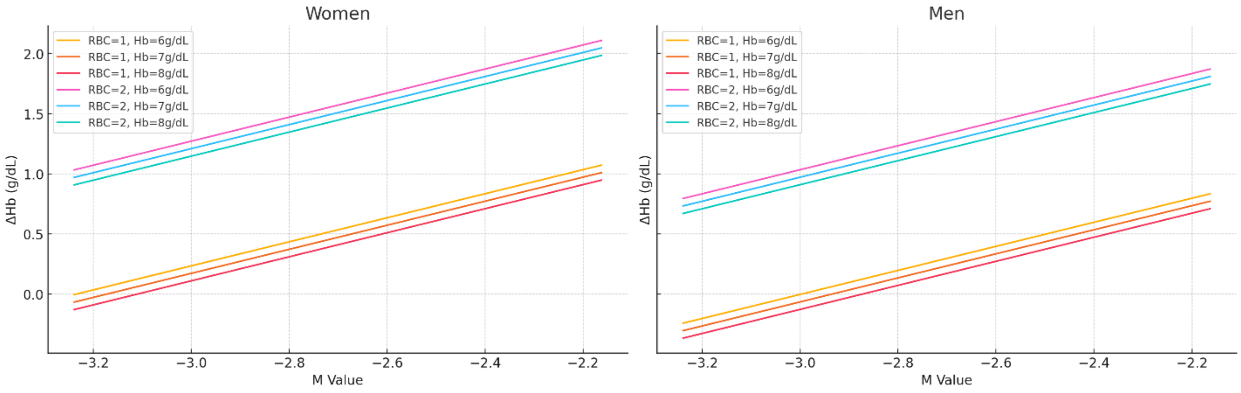

C

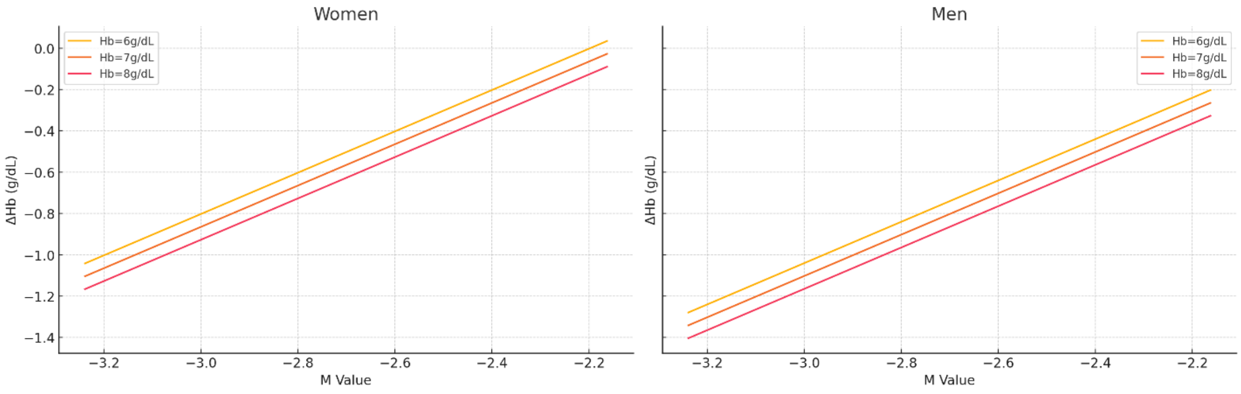

D

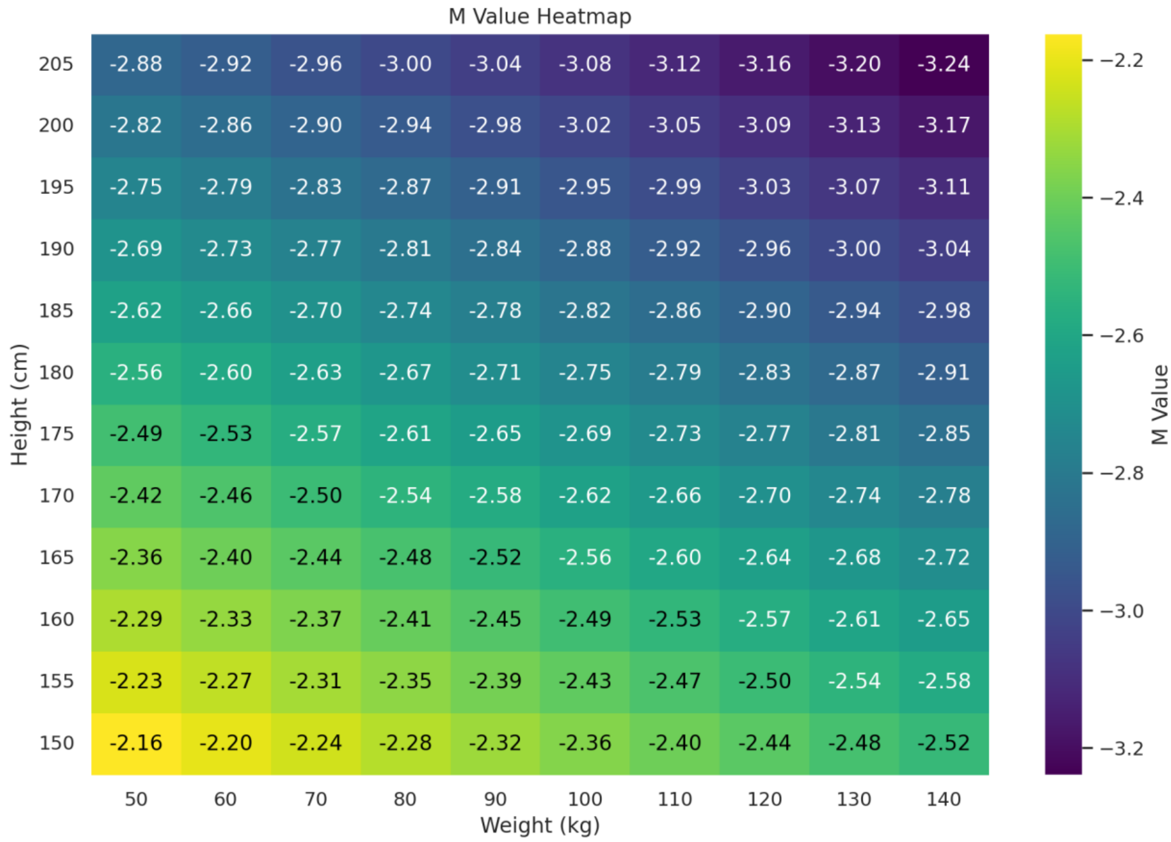

Suppl. Fig. 10. Predicted Hb increase after RBC transfusion derived from the linear regression model for the increase of Hb after transfusion (figure 3A).
Calculated with following assumptions: Crea=1mg/dl, NT-pro-BNP=100 ng/l and systolic blood pressure = 120 mmHg. With the following equation:

Eq. 10.1: $$\Delta Hb = 2.536 - 0.1310 \cdot \frac{height}{10} - 0.03954 \cdot \frac{weight}{10} - 0.2375 \cdot sex - 0.06223 \cdot Hb\,pre + 1.037 \cdot RBC\,units - 0.1714 \cdot PLT\,units + 0.01186 \cdot \frac{RR\,sys}{10} + 0.06206 \cdot Crea + 0.001524 \cdot \frac{NT{-}pro{-}BNP}{100}$$

For an easier handling of the graphs A-C we defined a factor M that represents the values for height and weight as follows:

Eq. 10.2: $$M = -0.1310 \cdot \frac{height}{10} - 0.03954 \cdot \frac{weight}{10}$$

(A-C) linear dependence of Hb from the multiple linear regression model in dependence from *M (see eq. 10.2 and D)*. Men and women, different Hb values without co-administration of PLT, AST negative; (B) Men and women, different Hb values with co-administration of PLT; (C) Only PLT units were transfused. (D) *M* can be derived from height and weight multiplied by the estimates (see equation 10.1).

Calculations and figures were created by ChatGPT 4.0.

Supplemental figure 11

A

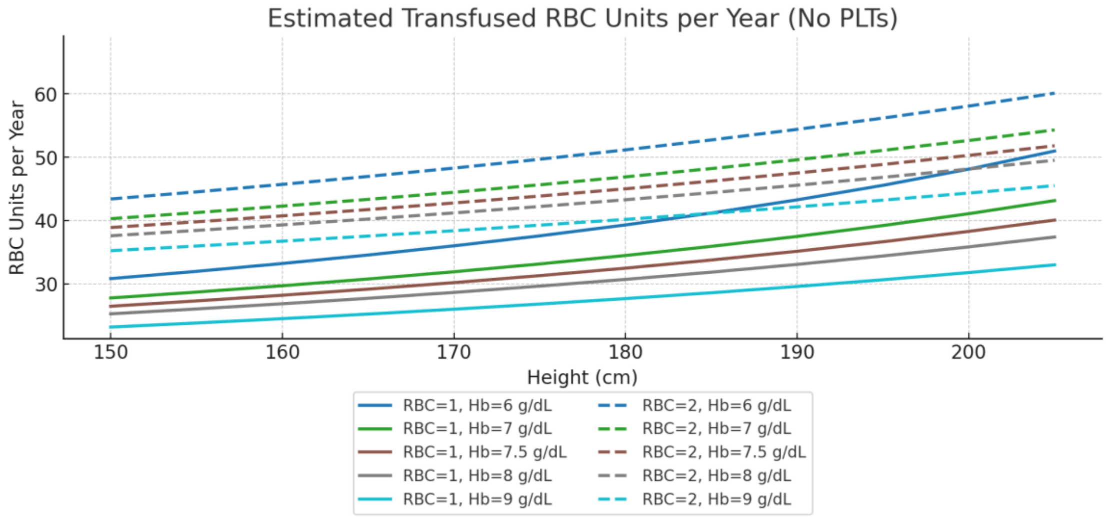

B

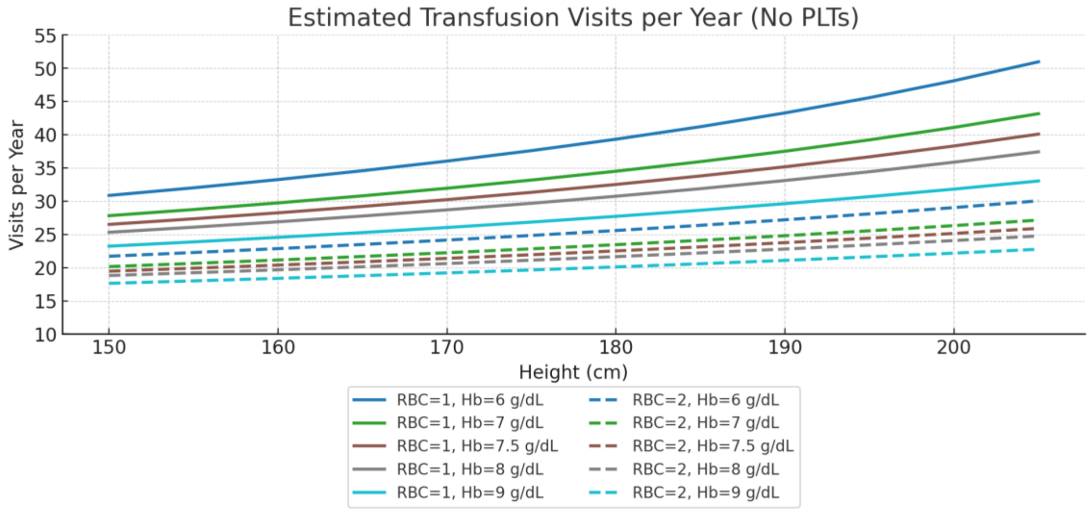

Suppl. Fig. 11 Transfused RBC units (A) and visits per year (B) by height and different pre-transfusion Hb levels without concomitant administration of PLTs.
Values were calculated as follows: *Time to next transfusion* was derived from linear regression model (**fig. 2C**)

Eg. 11.1:

$$Time\ to\ next\ transfusion\ (days) = 11.39 - 0.8485 \cdot \frac{height}{10} + 4.108 \cdot Hb\ pre - 7.558 \cdot \frac{Neu}{100} + 20.67 \cdot Reti\ post + 4.842 \cdot RBC\ units - 3.404 \cdot PLT\ units - 2.812 \cdot Hb\ next\ transfusion - 0.03595 \cdot \frac{ferritin}{100}$$

For Neu (neutrophils) =2.5 G/l, Reti post (reticulocytes after transfusion) 52980/µl (in case of one RBC unit) and 60000/µl (in case of 2 RBC units), Hb pre = Hb next transfusion, no co-administration of PLTs and ferritin=1000µg/l. Then we divided 365 days by *Time to next transfusion* to estimate the amount RBC units or the visits per year. (A) RBC units transfused per year for different pre-transfusion Hb levels by height. (B) Visits per year by different pre-transfusion Hb levels by height. Calculations and visualisation were done by ChatGPT 4.0.

Supplemental figure 12

A

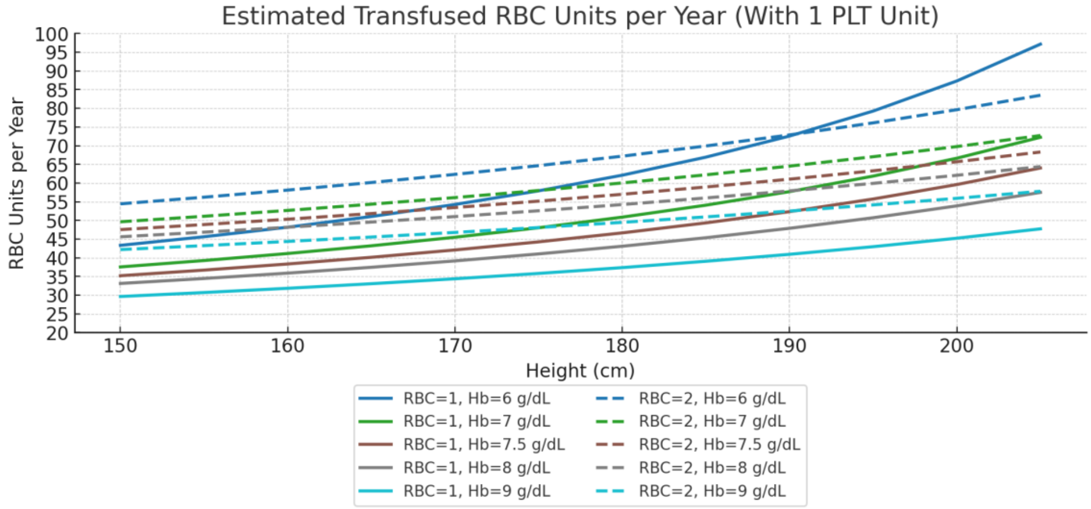

B

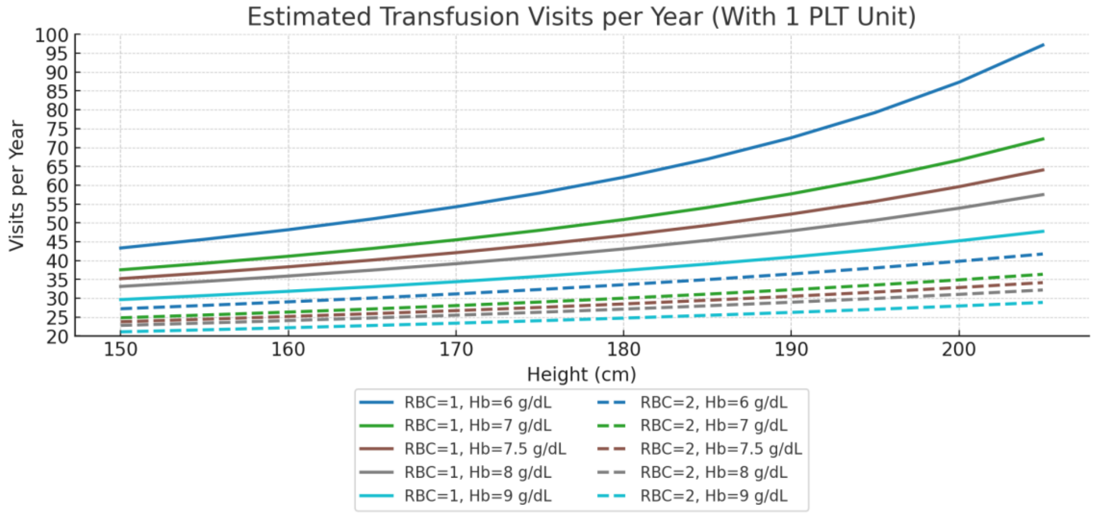

Suppl. fig. 12. Transfused RBC units (A) and visits per year (B), with concomitant transfusion of PLTs . For assumptions and calculations see suppl. Fig 11. Calculations and visualisation were done by ChatGPT 4.0.

Supplemental figure 13

A

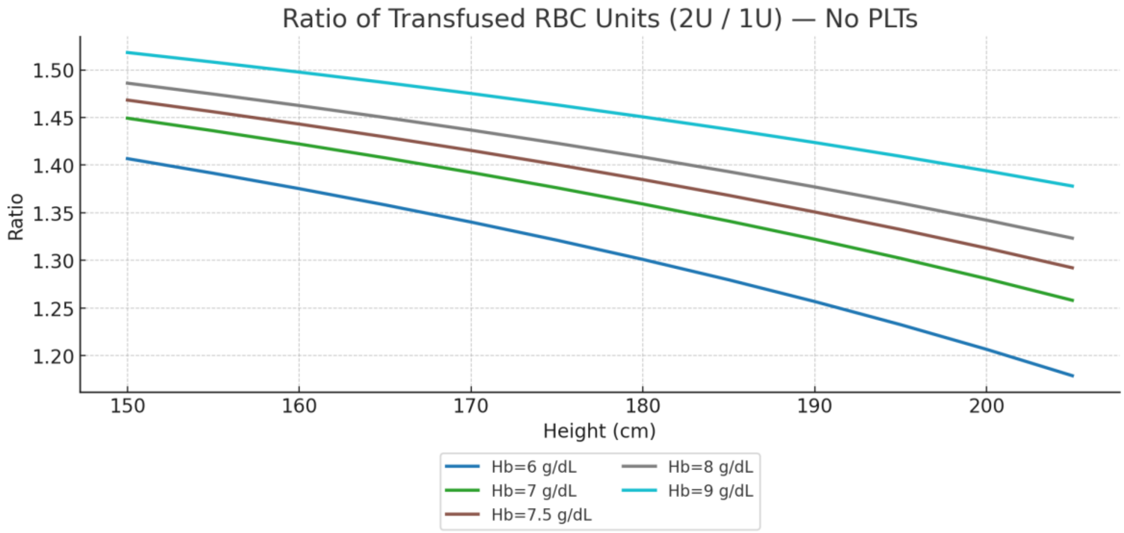

B

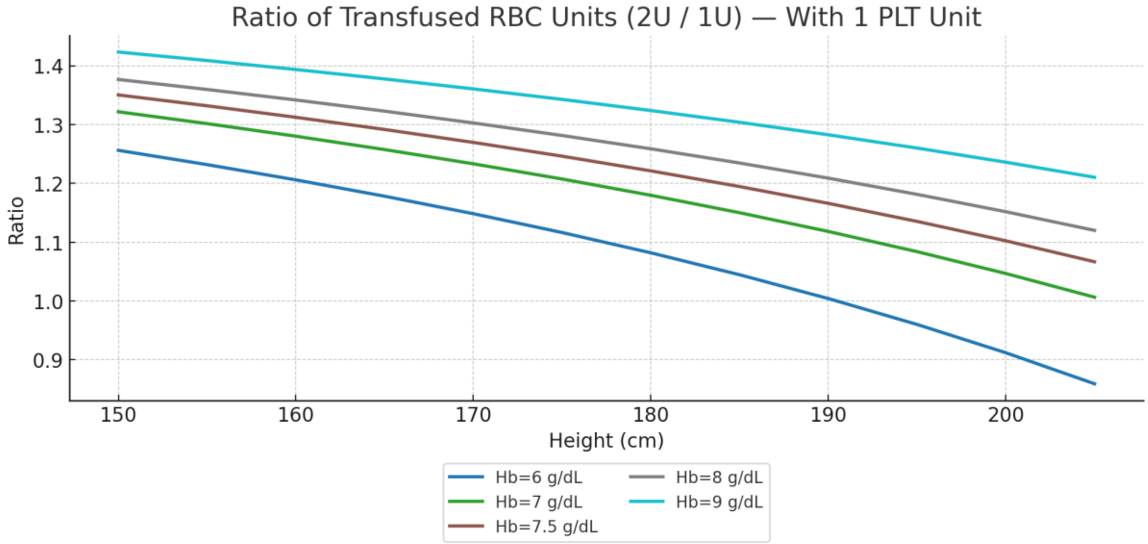

Suppl. fig. 13. Ratios of transfused units per years of a two unit versus a one unit strategy for different pre-transfusion Hb levels without PLT (A) and with PLT co-administration (B).
Values were calculated as follows: Curves for two unit strategy were divided by the curves for the one unit strategy and the ratio is displayed on the Y-axis.

Without PLT			With PLT		
**Hb (g/dl)**	**Height (cm)**	**Unit Savings (%)**	**Hb (g/dl)**	**Height (cm)**	**Unit Savings (%)**
6.0	150	**-28.1%**	6.0	150	**–21.3%**
6.0	170	**-21.9%**	6.0	170	**–13.0%**
6.0	205	**-12.3%**	6.0	205	**+17.6%**
7.5	150	**-31.0%**	7.5	150	**–25.0%**
7.5	170	**-27.0%**	7.5	170	**–20.0%**
7.5	205	**-20.6%**	7.5	205	**–15.0%**
9.0	150	**-33.3%**	9.0	150	**–27.0%**
9.0	170	**-30.1%**	9.0	170	**–24.0%**
9.0	205	**-24.8%**	9.0	205	**–18.7%**

Calculations and visualisation were done by ChatGPT 4.0.

Supplemental figure 14

A

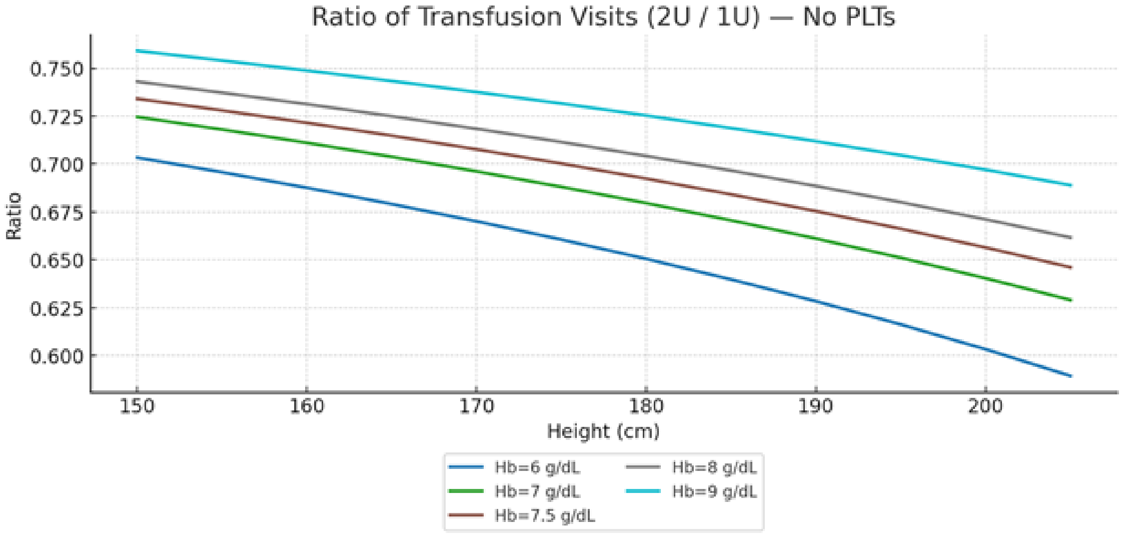

B

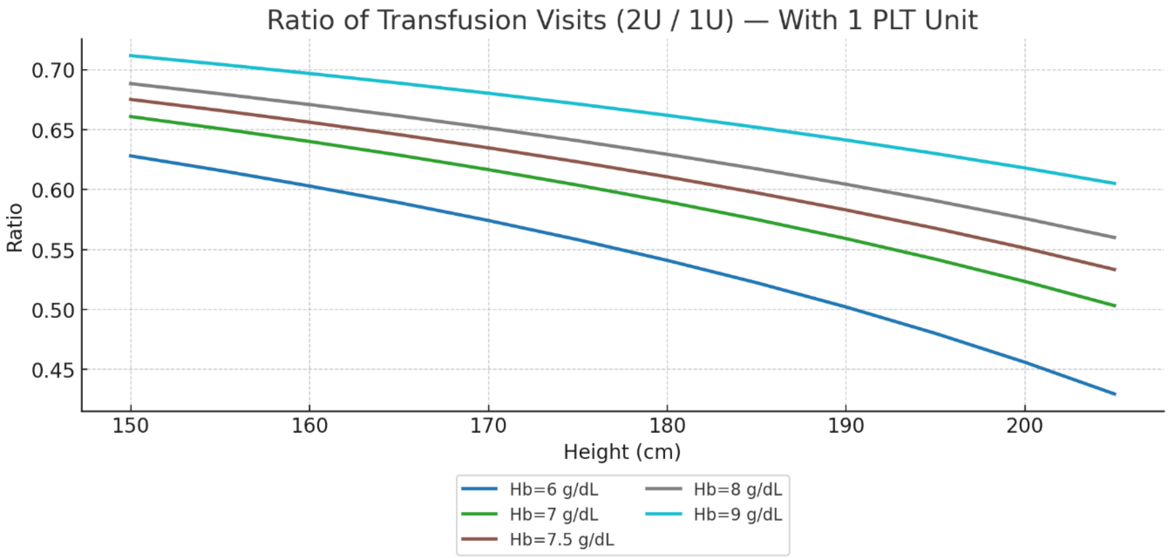

Suppl. fig. 14. Ratios of transfusion visits per year of a two unit versus a one unit strategy for different pre-transfusion Hb levels without PLT (A) and with PLT co-administration (B).
Values were calculated as follows: Curves for two unit strategy were divided by the curves for the one unit strategy and the ratio is displayed on the Y-axis.

Calculations and visualisation were done by ChatGPT 4.0.

Supplemental Methods

Red cell concentrates and Laboratory values

During August 2018 we changed the additive solution from SAG-M to PAGS-M. PAGS-M (Natriumchloride, Mannitol, Glucose-Monohydrat, Adenin, Guanosin, Natriumdihydrogenphosphat-Dihydrat, Natriummonohydrogenphosphat-Dihydrat, Aqua ad iniectabilia) (non-irradiated PEI.H.02408.01.1 or irradiated PEI.H.00944.01.1) . Overall 97.5 of units contained PAGS-M. Quality controls for 2021–2022 reported a mean hemoglobin content of 55.6 ± 10.9g/unit for irradiated or non-irradiated products (n=8147). Before August 2018 RBC products with the additive solution SAG-M were used.

A blood cell count was performed at each visit. Post-transfusion counts were taken immediately after RBC transfusion or 15 minutes after platelet (PLT) transfusion, using a XN-1000 analyser (Sysmex, Norderstedt, Germany). Determination of ferritin, creatinine with calculated glomerular filtration rate (GFR) and NT-pro-BNP was performed at the first visit and every six months thereafter, with values carried over to subsequent visits until updated. The values were then attributed to the following visits until new values were available.

Calculation of savings by a single RBC unit strategy

According to figure 4A (mean time to next transfusion) and B (mean of time to last and to next transfusion) the savings of RBC were calculated as Follows:

Eq 14.1: $$Visits\ year = \frac{365\ \text{days}}{\text{Interval}}$$

Eq 14.2: $$RBC\ units\ per\ year = \frac{365\ \text{days} * \text{units}}{\text{Interval}}$$

	1 unit	2 unit	Difference
**Figure 4 A**			
**Interval**	9.8 days	13.8 days	- 4 days
**Visits per year**	37.2 (140.9%)	26.4 (100%)	+40.9%
**Units per year**	37.2 (70.3%)	52.9 (100%)	-29.7%
**Figure 4 B**			
**Interval**	8.6 days	14.7 days	- 6.1 days
**Visits per year**	42.4 (171.0%)	24.8 (100%)	+ 71.0%
**Units per year**	42.4 (85.3%)	49.7 (100%)	- 14.7%

Composition of Cohort: Exclusion of patients with hemoglobinopathies, gastrointestinal bleeding

The indication of RBC transfusion in patients with hemoglobinopathies or gastrointestinal bleeding takes different measures into account like suppression of ineffective erythropoiesis and to provide a buffer of Hb for chronic bleeding. Transfusion strategies and the courses of Hb in these diseases might therefore differ from other anemias and are therefore excluded in analysis were this might be a relevant factor.

Verification of model-based results

To validate the analyses performed by ChatGPT, we recalculated representative scenarios from **supplemental figures 10–14** in Excel using the regression equations (Eqs. 10.1, 10.2, 11.1). These included ΔHb predictions (**supplemental figure 10**), transfusion intervals and derived annual RBC units/visits (**supplemental figures 11–12**), and ratios of 2-unit vs. 1-unit strategies (**supplemental figures 13–14**) for males and females with different body sizes and Hb levels. For each scenario, at least three representative patient constellations were recalculated. Because the underlying models are linear, such checks are sufficient to confirm overall validity. The recalculated values closely matched the plotted curves, with only rounding-level differences, confirming reproducibility of the results.

Multiple logistic regression

The Odds ratio was calculated by multiple logistic regression with a Main effects model, Reference levels for categorical variables were absence of conditions or female sex. Confidence intervals (95%) and P-values were calculated. For classification and prediction Area under ROC curve and classification table was used and a Pseudo R-Squared was calculated (**supplemental figure 6**). Variables were considered in the models when they were statistically significant.

Multiple linear regression models

A Main effects model was used. Reference levels for categorical variables were absence of conditions or female sex. Confidence intervals (95%) and P-values were calculated. The model was developed by including variables with potential influence to the corresponding outcome. The variables that were tested are listed in the **supplemental tables (4-7)**. The aim was to establish a simple model. First a model was tested with all variables that might have a meaningful contribution to the outcome variable. Combinations with error messages from Prism according to interaction were excluded. Then variables were further excluded the model was checked for r^2 and tested in other combinations. Examples for combinations are listed in the supplemental tables (3-6), p-values indicate the inclusion of the example models. Variables that were also tested but not stated in an example are marked by "-". Models that were finally chosen are displayed in green.

Missing values were substituted for creatinine, ferritin and pro-NT-BNP. Because of the importance of height and weight we decided not to substitute missing values for these variables.

Supplemental Table 4: ΔHb_0

Variables	1	2	3	4	5
Intercept	<0.0001	<0.0001	<0.0001	<0.0001	<0.0001
ST	0.8920	-	-	-	-
A-NOS	0.2154	-	-	-	-
BD	0.0385	-	-	-	-
Heart disease	0.2063	-	-	-	-
Fatigue	0.0799	0.9650	-	-	-
Dyspnea	0.0027	0.0037	0.0018	-	-
Height [10cm]	<0.0001	<0.0001	<0.0001	<0.0001	<0.0001
Weight [10kg]	<0.0001	<0.0001	<0.0001	<0.0001	<0.0001
Age [10years]	0.0093	0.0003	0.0005	-	-
Sex [m]	<0.0001	<0.0001	<0.0001	<0.0001	<0.0001
Hb pre [g/dl]	0.0004	<0.0001	<0.0001	<0.0001	<0.0001
Reti pre [10^6/µl]	0.2165	-		-	-
WBC pre [10^3/µl]	0.2018	-	-	-	-
ANC [10/µl]	0.0030	<0.0001	<0.0001	<0.0001	-
PLT [1000/µl]	0.6560	-	-	-	-
AST pos*	0.1757	-	-	-	-
RBC [unit]	<0.0001	<0.0001	<0.0001	<0.0001	<0.0001
PLT [unit]	<0.0001	<0.0001	<0.0001	<0.0001	<0.0001
RR diast [10mmHg]	0.0291	0.3421	-	-	-
RR sys [10mmHg]	0.0003	0.0049	0.0041	0.0205	0.0055
Pulse [10/min]	0.3879	0.0202	0.0169	-	
RBC last given	0.0052	<0.0001	<0.0001	-	-
Time since last RBC [day]	0.4611	-	-	-	-
Creatinine [mg/dl]	0.0012	<0.0001	<0.0001	<0.0001	0.0002
NT-pro-BNP [1000ng/l]	0.0002	<0.0001	<0.0001	<0.0001	<0.0001
Ferritin [1000µg/l]	0.3852	-	-	-	-
Chelator therapy	0.144	-	-	-	-
n	1650	2720	2720	2723	2742
R^2	0.6346	0.8522	0.8522	0.8499	0.8468

Rows in table: n=4613

*AST was investigated by using different categories. Here the 2 categorical approach is shown (0=negative, 1=positive) another version included 3 categories (0=negative, 1=positive and 2=not performed, yielded p=0.8439. It was not used because the category 2 defined a group with RBC=0 and PLT≥1, defining a separate group, were $\Delta Hb_0 \leq 0$ because no RBC are given and the estimates for PLT units and RBC were biased. Model 5 was finally preferred to simplicity (*ANC [10/µl]* skipped) and suspicion against *RBC last given (*see Model 4*)* as a meaningful variable in this context.

Supplemental table 5: ΔHb_t next visit

Variables	1	2	3	4	5
Intercept	0.2050	0.6167	0.9456	0.7399	0.7555
A-NOS	0.0438	0.0460	0.0423	0.0665	0.0507
ST	<0.0001	0.0008	0.0010	0.0024	0.0010
BD	<0.0001	0.0002	0.0002	0.0005	0.0002
Time to next visit [days]	<0.0001	<0.0001	<0.0001	<0.0001	<0.0001
Height [10cm]	0.0002	0.0009	<0.0001	<0.0001	<0.0001
Weight [10kg]	0.0053	0.3056	-	-	-
Age [10years]	0.0036	0.0014	0.0018	0.0018	0.0019
Sex [m]	0.0014	<0.0001	<0.0001	<0.0001	<0.0001
WBC	0.0695	-	-		-
ANC [100/µl]	0.1481	-	-	-	-
PLT [1000/µl]	0.0944	-	-	-	-
Hb post [g/dl]	<0.0001	0.0042	0.0029	0.0009	0.0028
Reti post [10^6/µl]	<0.0001	<0.0001	<0.0001	-	<0.0001
RBC [unit]	0.0039	0.0014	<0.0001	0.0002	0.0012
PLT [unit]	<0.0001	<0.0001	<0.0001	<0.0001	<0.0001
Creatinine [mg/dl]	0.0513	-	-	-	-
NT-pro-BNP [1000ng/l]	0.8171	-	-	-	-
Ferritin [1000µg/l]	<0.0001	<0.0001	<0.0001	<0.0001	<0.0001
Chelator therapy	0.1860	-	-	-	-
Hb pre [g/dl]	<0.0001	-	-	-	-
Reti pre [106/µl]	-	-	-	<0.0001	-
AST 3[not performed]	0.0481	0.2350-	-	0.0835	0.2283
AST 3 [positive]	0.0281	0.0415	-	0.1279	0.0499
					-
n	1904	1932	1933	1990	1933
R2	0.4767	0.4733	0.4716	0.4708	0.4730

Rows in table: n=3175

Variables that might have an effect on ΔHb_0 were initially included, Variables that are dependent (e.g. RBC in combination with Hb pre and post) were not tested at the same time (row 1). Non-significant variables were excluded in the next steps. Alternative combinations of variables that are depended were tested. Finally, variant 5 was chosen over 4 because the methodologic considerations (see table 3).

Supplemental table 6: Time to next RBC

Variables	1	2	3	4	5	6	7	9
Intercept	0.0345	0.0090	<0.0001	<0.0001	0.0002	0.0616	0.0019	0.034
A-NOS	0.0785	0.1055	-	-	-	-	-	-
ST	0.2313	0.3851	-	-	-	-	-	-
BD	0.4793	0.6929	-	-	-	-	-	-
Height [10cm]	<0.0001	<0.0001	<0.0001	<0.0001	<0.0001	0.0295	-	0.0358
Weight [10kg]	0.7141	0.7826	-	-	-	-	-	-
Sex [m]	0.3946	-	-	-	-	-	-	-
Hb pre [g/dl]	<0.0001	<0.0001	<0.0001	<0.0001	<0.0001	-	-	0.0323
WBC pre [10^3/µl]	0.3361	-	-	-	-		-	-
ANC [10/µl]	0.0372	<0.0001	<0.0001	<0.0001	<0.0001	0.0021	0.0005	-
PLT [1000/µl]	0.6962	-	-	-	-	-	-	-
Hb post [g/dl]	-	-	-	-	-	<0.0001	<0.0001	<0.0001
Reti post [10^6/µl]	<0.0001	<0.0001	<0.0001	<0.0001	<0.0001	<0.0001	0.0005	<0.0001
RBC [unit]	<0.0001	<0.0001	<0.0001	<0.0001	<0.0001	-	-	-
AST [pos.]*	0.0008	<0.0001	0.0001	0.0001	-	-	-	-
PLT [unit]	<0.0001	<0.0001	<0.0001	<0.0001	<0.0001	<0.0001	<0.0001	<0.0001
Chelator therapy	0.0266	0.0441	0.0816	-	-	-		
Hb at next RBC transfusion [g/dl]	0.3602	<0.0001	<0.0001	<0.0001	<0.0001	<0.0001	<0.0001	<0.0001
Hb at last RBC transfusion [g/dl]	0.3720	-	-	-	-	-	-	-
RBC last given [units]	0.1831	-	-	-	-	-	-	-
Creatinine [mg/dl]	0.2620	-	0.0472	-	-	-	-	-
Ferritin [µg/l]	<0.0001	<0.0001	<0.0001	<0.0001	<0.0001	<0.0001	-	-
NT-pro-BNP-1 [1000ng/l]	0.0866	0.0289	0.0449	-	-	-	0.0045	0.0019
						-	-	
n	995	1049	1050	1060	1063	1063	1634	1069
R2	0.5064	0.4952	0.4875	0.4881	0.4804	0.4817	0.4248	0.4856

Rows in table: n=1764

*AST was performed at all included visits (only visits were included with RBC transfusions)

Supplemental table 7: Δ heart rate

Variables	1	2	3	4
Intercept	0.0084	<0.0001	<0.0001	<0.0001
Other	-		-	-
A-NOS	-	-	-	-
BD	-	-	-	-
Heart disease	-	0.2436	-	-
Fatigue [not present]	-	-	-	-
Dyspnea	-	-	-	-
Height [10cm]	<0.0001	0.0149	0.0184	0.0207
Weight [10kg]	0.1172	0.0001	0.0002	0.0003
Sex [m]	0.8585	<0.0001	<0.0001	0.0001
Hb pre [g/dl]	0.7083	0.1773	0.1601	-
ANC [10/µl]	-	-0.0101	0.0054	0.0049
Hb post [g/dl]	-	-	-	-
RBC [unit]	<0.0001	0.0002	0.0001	0.0003
PLT [unit]	0.0604	0.0001	0.0001	<0.0001
AST [pos.]	-	-	-	-
Creatinine [mg/dl]	<0.0001	<0.0001	<0.0001	<0.0001
RR sys [10mmHg]*	0.556	0.0028	0.0036	0.0039
Pulse [10 beats/min]*	-	<0.0001	<0.0001	<0.0001
NT-pro-BNP 1[1000ng/l]	0.0152	<0.0001	<0.0001	<0.0001
RBC/BV [units/l]	-	-	-	-
RR diast [10mmHg]*	-	-	-	-
BV [l]	-	-	-	-
	-	-		
n	2718	2716	2716	2698
R2	0.05503	0.4155	0.4152	0.4143

Rows in table: n=4613

*pre transfusion

Starting point was the model from table 3.

Literature

1. Reikvam H, Prowse C, Roddie H, Heddle NM, Hervig T, collaborative B. A pilot study of the possibility and the feasibility of haemoglobin dosing with red blood cells transfusion. Vox Sang. 2010;99(1):71-6.
